# Multi‐Dimensional Acoustic Cascaded Holographic Encryption with Instantaneous Visual Decryption via Particle Manipulation

**DOI:** 10.1002/advs.202516151

**Published:** 2025-12-19

**Authors:** Qin Lin, Feiyan Cai, Yunqing Liu, Zejiao Zhou, Jiayang Li, Rujun Zhang, Hongpeng Chen, Hairong Zheng, Huailing Zhang

**Affiliations:** ^1^ School of Biomedical Engineering, Dongguan Key Laboratory of Medical Electronics and Medical Imaging Equipment, Songshan Lake Innovation Center of Medicine & Engineering Guangdong Medical University Dongguan 523808 P. R. China; ^2^ Paul C. Lauterbur Research Center for Biomedical Imaging, Shenzhen Institutes of Advanced Technology, The Key Laboratory of Biomedical Imaging Science and System Chinese Academy of Sciences Shenzhen 518055 P. R. China

**Keywords:** acoustic holograms, deep learning, field visualization, information encryption, particle manipulation

## Abstract

Acoustic holograms have emerged as valuable tools for information encryption owing to their versatile capabilities in sound field manipulation. However, current acoustic hologram‐based encryption schemes suffer from two key drawbacks: first, they predominantly rely on encoding information into amplitude and/or phase profiles, limiting encryption dimensionality and security; second, their decryption processes depend on time‐consuming mechanical scanning or complex signal processing, hindering real‐time performance and practical applicability. Herein, a compact acoustic encryption device is demonstrated by integrating multi‐dimensional multiplexed cascaded acoustic holography with particle manipulation. Beyond phase profiles, this device further employs the distance and in‐plane rotation angle between cascaded holograms as additional secret keys to enhance encryption dimensionality and security. Notably, its decryption process is achieved through particle patterning within tens of seconds, eliminating the need for auxiliary complex instrumentation to visually reveal encrypted images. To achieve this, a physics‐driven neural network‐based inverse design scheme is developed to optimize the cascaded holograms. Experimental validation through 1D, 2D, and 3D encryption tests confirms the device's practicality, demonstrating enhanced encryption security and instantaneous visual decryption. Owing to its advantages of compact size, instantaneous visual decryption, and enhanced security, this device has great potential in acoustic encryption, cell/tissue engineering, and dynamic holographic displays.

## Graphical abstract

Acoustic holograms act as key tools for information encryption, yet current schemes limit encryption dimensionality/security and require time‐consuming decryption. A compact device integrates multi‐dimensional cascaded acoustic holography with particle manipulation, employs extra secret keys, enables rapid decryption, and is validated via 1D/2D/3D tests, holding promise for acoustic encryption, cell/tissue engineering, and dynamic holographic displays.

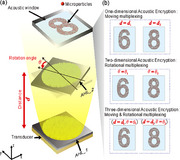

## 1 Introduction

As digital data permeates every aspect of modern society, from critical infrastructure operations to personal communication, information security has ascended to an unprecedented level of importance. Extensive efforts have been devoted to developing advanced encryption methods to improve information security.^[^ ^1^, ^2^, ^3^, ^4^, ^5^, ^6^, ^7^, ^8^, ^9^, ^10^, ^11^, ^12^, ^13^, ^14^, ^15^, ^16^, ^17^, ^18^, ^19^ ^]^ Among these, encryption methods based on electromagnetic (EM) waves have seen rapid and widespread development, as they offer the advantages of high‐speed data transmission, good compatibility with existing wireless communication infrastructure, high capacity, and high security. However, they are less effective in environments with electromagnetic shielding, and underwater, due to their intrinsic severe attenuation and scattering characteristics.^[^ ^11^, ^13^, ^18^, ^20^, ^21^ ^]^ Conversely, as acoustic waves are not affected by electromagnetic fields, acoustic waves‐based devices are well‐equipped to work in electromagnetic‐shielded conditions. Moreover, compared to EM waves, acoustic waves exhibit lower attenuation and scattering underwater, which allows them to penetrate deeper, enabling more reliable long‐range underwater communication. Consequently, acoustic waves become a crucial alternative for implementing information encryption technologies in environments where EM waves‐based encryption methods are unsuitable.

To date, while a considerable number of encryption methods based on EM waves have been developed for information encryption, those using acoustic waves, however, have received relatively limited exploration. In particular, acoustic holograms have emerged as one of the most promising techniques for achieving acoustic information encryption, primarily owing to their exceptional capability in acoustic field modulation and abundant degrees of freedom (DOFs).^[^ ^11^, ^13^, ^18^, ^19^ ^]^ Conventionally, acoustic holograms are typically implemented using active phased‐array transducers.^[^ ^22^, ^23^ ^]^ These active phased‐array transducers enable the generation of flexible and reconfigurable acoustic fields, which can be exploited for information encryption. For instance, the acoustic vortex beams with varying orbital angular momentum (OAM) topological charges, generated by an active transducer array, have been demonstrated to enhance data transmission rates and information capacity.^[^ ^18^ ^]^ In this study, encrypted information is first represented using the ASCII binary protocol. It is then encoded and transmitted via superimposed multiplexed acoustic vortex beams with OAM charges ranging from ‐4 to +4. Finally, an inner‐product algorithm and a sensor array are employed to decode the encrypted information. Moreover, an acoustics‐based wireless infrastructure has been developed for parallel acoustic communication using active phased‐array transducers.^[^ ^13^ ^]^ In this configuration, since the active phased‐array transducers can dynamically modulate multiple focal patterns, the information, whose pixels are pre‐shaped into ten binary sequences, can be flexibly and precisely encrypted into the amplitudes of ten foci using the amplitude‐shift keying method. This approach ensures minimal crosstalk and enhanced security. Nevertheless, the active phased‐array transducers suffer from poor scalability, high power consumption, and cumbersome calibration requirements.^[^ ^11^, ^19^, ^24^, ^25^, ^26^, ^27^, ^28^, ^29^, ^30^, ^31^ ^]^ These drawbacks severely restrict the spatial fidelity and number of DOFs in reconstructed acoustic fields. Hence, encryption methods based on active phased‐array transducers often need to reshape the encrypted image into sets of binary data for information encoding and transmission, resulting in limited information capacity. Recently, passive acoustic holograms have attracted considerable attention in the field of information encryption. Benefiting from 3D printing technology, these passive acoustic holograms boast high pixel density, enabling them to encrypt information with considerably higher spatial fidelity and a greater number of DOFs compared to traditional active phased‐array transducers.^[^ ^11^, ^19^, ^24^, ^25^, ^26^, ^27^, ^28^, ^29^, ^30^, ^31^ ^]^ For example, an acoustic encryption framework integrating an acoustic speaker array with a reusable acoustic meta‐key has been developed for information encryption.^[^ ^11^ ^]^ By leveraging the precise acoustic field modulation capabilities of passive acoustic meta‐surfaces, information can be encrypted in image form and retrieved with high fidelity and security. Furthermore, an amplitude‐phase dual‐channel encrypted acoustic meta‐hologram (DrEAM) has been successfully realized by utilizing passive acoustic meta‐surfaces capable of simultaneous and decoupled amplitude and phase modulation.^[^ ^19^ ^]^ In contrast to the previous work, DrEAM incorporates a phase holographic image as an additional DOF for information encryption, thereby enhancing both information capacity and security. Very recently, a secret‐sharing acoustic encryption method has been implemented using cascaded acoustic holographic plates (AHPs), where the encrypted information is spatially divided into multiple AHPs (secret keys).^[^ ^6^ ^]^ The encrypted information can only be deciphered when the requisite secret keys are properly stacked along the diffraction path. Under this arrangement, a single stolen secret key can only reconstruct the authentication image without revealing any part of the secret information, thereby further enhancing both information security and capacity significantly. Nevertheless, despite the remarkable progress in enhancing information security and capacity, these acoustic encryption methods based on the passive acoustic holograms still have limitations. Specifically, the information is merely encoded into the amplitude and/or phase profiles of acoustic holograms, which poses latent information disclosure risks for non‐authorized parties to decrypt concealed information. Moreover, the decryption procedure of these methods usually requires additional complex instruments or sophisticated algorithms for field scanning and data decoding, which not only impedes the retrieving efficiency of encrypted information but also presents substantial challenges in physical implementation for real‐world applications.^[^ ^20^, ^21^ ^]^ Therefore, it is imperative to develop novel acoustic encryption methods that can encrypt information with more additional DOFs while decrypt it without sensor scanning or post processing for establishing a more secure and compact information encryption system.

Recently, previous studies have attempted to utilize the sound velocity of the fluidic medium^[^ ^31^ ^]^ or a detachable  coupling layer^[^ ^32^ ^]^ as additional DOFs to encode multiple images, while leveraging colored polydimethylsiloxane (PDMS) particles for acoustic field visualization in microfluidic applications. However, these techniques have not yet been extended to acoustic encryption. Inspired by these works, we propose and experimentally demonstrate a compact acoustic encryption device, termed as the visual and multi‐dimensional cascaded holographic acoustic encryption (VMD‐CHAE) device. This device integrates the multi‐dimensional multiplexing cascaded acoustic holography and particle manipulation, enabling the simultaneous realization of multi‐dimensional information encryption and instantaneous visual holographic decryption. Leveraging the capabilities of multi‐dimensional multiplexing cascaded acoustic holography, the device further exploits the distance and in‐plane rotation angle introduced by two cascaded acoustic holographic lenses (AHLs), in addition to the phase profiles, as two additional DOFs for information encryption. This imposes more stringent conditions for decrypting secret information, thereby substantially enhancing the design flexibility and information security. Notably, the decryption process is achieved through rapid particle patterning, eliminating the need for auxiliary instrumentation or machinery to reveal the visual information of the encrypted holographic images. This markedly improves decryption efficiency while substantially reducing the overall system size and complexity. To achieve such novel functionalities, the primary challenges lie in the significant crosstalk and efficiency loss caused by cascaded AHLs.^[^ ^33^, ^34^, ^35^, ^36^ ^]^ Given that deep learning methods have demonstrated significant improvements in both the reconstruction accuracy and efficiency of acoustic hologram design,^[^ ^33^, ^34^, ^37^, ^38^, ^39^, ^40^, ^41^, ^42^ ^]^ we further develop a physics‐driven neural network‐based inverse design scheme to carefully design the phase profiles of two cascaded AHLs. By employing this physics‐driven neural network‐based inverse design scheme, acoustic energy can efficiently transmit through two cascaded AHLs and concentrate precisely on the positions and patterns of encrypted holographic images. This facilitates accurate and robust particle patterning, enabling rapid, high‐quality holographic visual decryption. We numerically and experimentally validate the performance of our VMD‐CHAE device via 1D, 2D, and 3D acoustic encryption experiments. These demonstrations highlight the device's capabilities in enhanced secure encryption and high‐quality instantaneous visual decryption. The VMD‐CHAE device overcomes the limitations of existing acoustic hologram‐based encryption schemes, thereby paving the way for advancements in a wide range of fields including acoustic encryption, underwater communications, volumetric displays, dynamic particle manipulation, tissue engineering, and medical ultrasound.

## 2 Results

### 2.1 Working Principle

**Figure 1:**
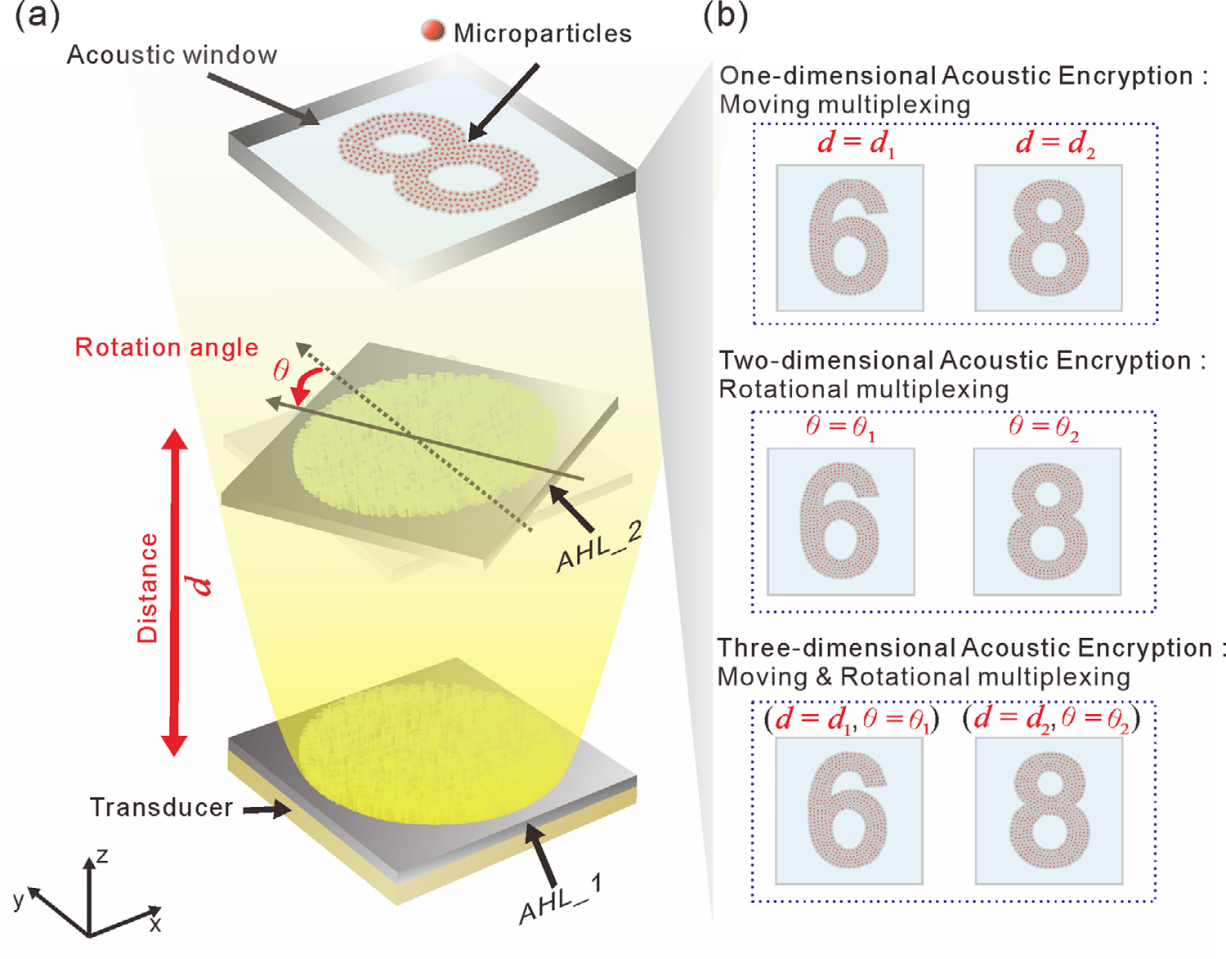
Conceptual illustration of multi‐dimensional acoustic encryption and visual encryption based on our VMD‐CHAE device. a) Schematic illustration of the structure and acoustic wave propagation of our VMD‐CHAE device. When incident acoustic waves are modulated by the cascaded AHLs, different target acoustic fields can be generated within the acoustic window according to the combination of distance and in‐plane rotation angle between two cascaded AHLs for achieving multi‐dimensional acoustic encryption. Subsequently, microparticles within the acoustic window are rapidly patterned by the acoustic field, providing an intuitive and instantaneous visual decryption of the corresponding encrypted holographic image. b) Demonstrations of 1D, 2D, and 3D acoustic encryptions and visual encryption for two holographic images.

**Figure** **1** illustrates the working mechanism of our VMD‐CHAE device. As shown in Figure 1a, the device consists of two independent AHLs. The first AHL (AHL_1) is attached to the front surface of the transducer, while the second AHL (AHL_2) can be selectively cascaded with tunable distances or/and in‐plane rotation angles. The colored PDMS microparticles are randomly suspended on the water surface, which is positioned at the target plane within the acoustic window. Initially, the incident plane acoustic waves generated by the transducer are modulated by AHL_1. Subsequently, the modulated acoustic waves propagate through AHL_2, which is configured with a specific distance and/or in‐plane rotation angle. As the modulated acoustic waves enter the acoustic window, the first encrypted holographic image is visualized as a corresponding particle pattern within the acoustic window. This phenomenon occurs because, according to the acoustic radiation forces (ARFs) analysis (see Experimental Section for details),^[^ ^43^, ^44^, ^45^ ^]^ the colored PDMS microparticles with a negative acoustic contrast factor are trapped by ARFs toward regions of high acoustic field intensity, rapidly assembling into the shape of the corresponding encrypted holographic image. This dynamic particle self‐assembly process enables direct and rapid visual decryption of the encrypted holographic information. By varying the combinations of distances and in‐plane rotation angles between two AHLs, different phase delays are accumulated, allowing for the sequential visualization of other encrypted holographic images within the acoustic window.

The encrypted holographic images, visualized as particle patterns within the acoustic window, are determined by the combination of distance, in‐plane rotation angle, and the phase profiles of two cascaded AHLs. Only when two AHLs are appropriately cascaded with a correct distance or/and a correct in‐plane rotation angle, the design encrypted holographic image can be precisely decrypted and visualized as corresponding particle pattern within the acoustic window. Therefore, in addition to the phase profiles of two cascaded AHLs, the distance and in‐plane rotation angle can be further utilized as two additional design DOFs to encrypt encrypted holographic images, enabling multi‐dimensional acoustic encryption. For instance, two identical holographic images of the digits “6” and “8” can be encrypted using two distinct distances or two separate in‐plane rotation angles to achieve 1D or 2D acoustic encryption, respectively. Furthermore, 3D acoustic encryption can be realized by combining both the distance and in‐plane rotation angle as two additional design DOFs. Such a multi‐dimensional acoustic encryption technique enabled by the multi‐dimensional multiplexing cascaded acoustic holography provides greater flexibility and complexity for information encryption, thereby significantly enhancing design versatility and information security. By integrating the advantages of multi‐dimensional multiplexing cascaded acoustic holography and rapid particle manipulation, our VMD‐CHAE device simultaneously achieves multi‐dimensional information encryption and instantaneous visual decryption without the need for additional complex mechanical or electronic components.

### 2.2 Inverse Design of Cascaded Acoustic Holograms

**Figure 2:**
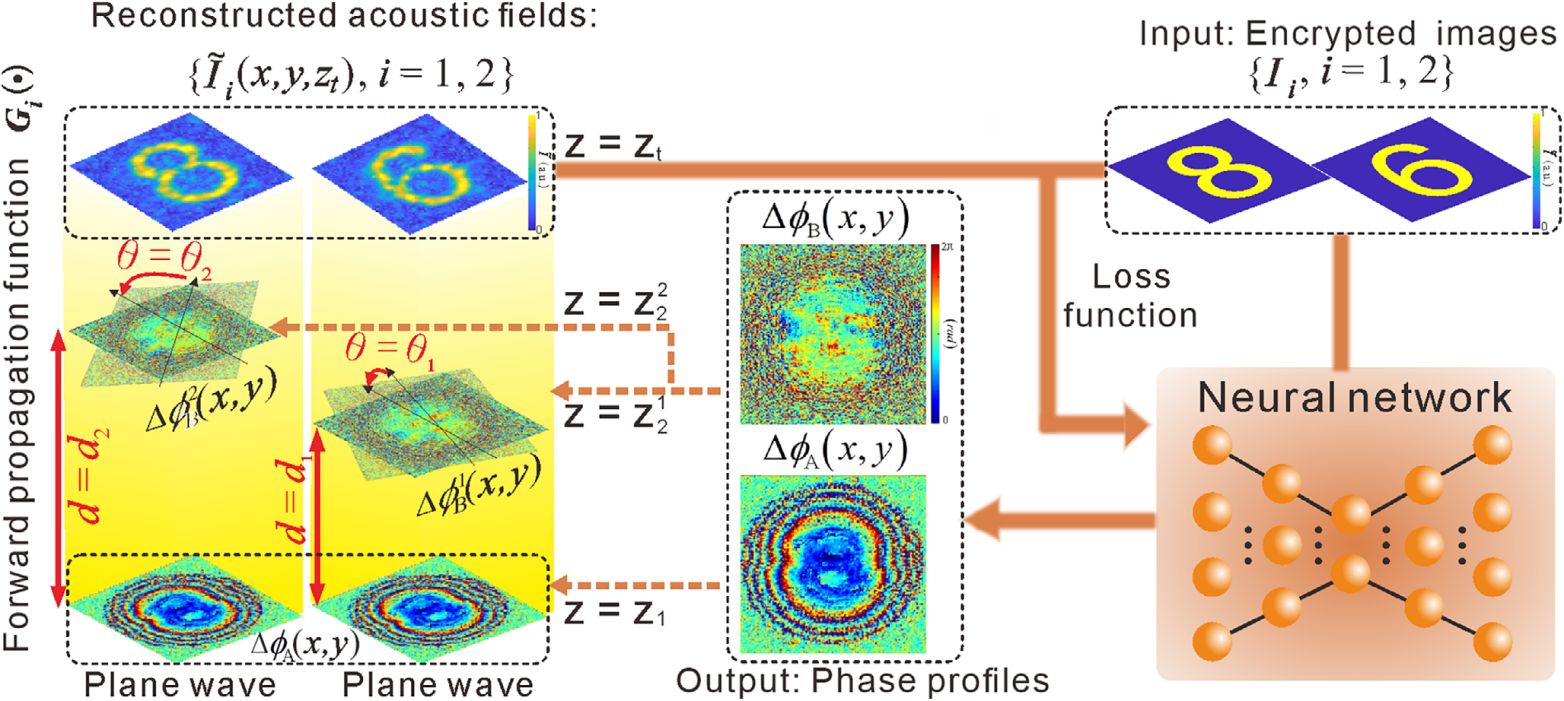
Schematic diagram of the physics‐driven neural network‐based inverse design scheme. The only inputs are multiple encrypted holographic images. The outputs are the optimized phase profiles of two cascaded AHLs, which are then used to numerically simulate the reconstructed acoustic intensity fields using forward propagation functions. The loss function between multiple encrypted holographic images and their corresponding reconstructed acoustic fields across all design combinations of distances and in‐plane rotation angles is used to optimize the neural network.

The design algorithm governing the phase profiles of the cascaded AHLs is pivotal in achieving high‐fidelity multi‐dimensional acoustic encryption and rapid visual decryption, as the particle manipulation performance is critically dependent on the quality of the reconstructed acoustic fields. To obtain high‐quality reconstructed acoustic fields, we propose a physics‐driven neural network‐based inverse design scheme to inversely optimize the phase profiles of two AHLs. This scheme integrates two key components: explicit forward propagation functions and a deep neural network architecture, as illustrated in **Figure** **2**. The primary objective of this scheme is to minimize the loss function between multiple encrypted holographic images and their corresponding reconstructed acoustic fields across all design combinations of distances and in‐plane rotation angles (d = d_i_,θ = θ_i_), i ∈ {1, 2, · · ·, N}, i.e.,

(1) $$R_{\theta^{*}}=\underset{\theta \in \Theta }{\arg\min}\frac{1}{N}\sum_{i=1}^{N}{\left\|\left({\tilde{I}}_{i}\left(x,y,z_{t}\right)-I_{i}\right)\right\|}^{2}$$

(2) $${\tilde{I}}_{i}\left(x,y,z_{t}\right)=G_{i}\left(\Delta \phi_{A}\left(x,y\right),\Delta \phi_{B}\left(x,y\right)\right)$$

where *R*_θ_ represents the mapping function of the deep neural network parameterized by weights and biases, ${\tilde{I}}_{i}\left(x,y,z_{t}\right)$ represents the *i* ^th^ reconstructed acoustic intensity field that is forward simulated from the phase profiles Δϕ_*A*_(*x*,*y*) and Δϕ_*B*_(*x*,*y*) of two AHLs using *G_i_* (·),*G_i_* (·)denotes the *i* ^th^ forward propagation function that is analytically modeled using angular spectrum diffraction theory^[^ ^30^, ^33^, ^34^, ^35^, ^37^, ^40^ ^]^ for the *i* ^th^ combination of distance *d* = *d_i_* and in‐plane rotation angle θ = θ_*i*_ (Section S1, Supporting Information), *I_i_* represents the *i* ^th^ encrypted holographic image. Notably, the proposed physics‐driven neural network‐based inverse design scheme eliminates the need for extensive training datasets of ground‐truth phase profiles, as the objective function Equation (1) directly optimizes the network parameters without explicit reliance on phase profile inputs. Instead, the interplay between the forward propagation functions *G_i_* (·) and the deep neural network *R*_θ_ enables the network to inherently capture the underlying physical priors *I_i_* through the optimization process. Furthermore, as U‐Net^[^ ^46^ ^]^ is a well‐established neural network architecture for image reconstruction problems, we utilize its symmetric encoder‐decoder structure with skip connections to construct the deep neural network *R*_θ_. Specifically, the network comprises an encoder path taking multiple design encrypted holographic images as input, a decoder path that outputs optimized phase profiles Δϕ_*A*_(*x*,*y*)and Δϕ_*B*_(*x*,*y*) of two AHLs, and multiple intermediate skip paths. The architecture employs four key module types: convolution blocks (3 × 3 convolutions + batch normalization + leaky ReLU), max pooling blocks (2 × 2 max pooling), up‐sampling blocks (2 × 2 transposed convolutions + batch normalization + leaky ReLU), and skip connection blocks.

The proposed physics‐driven neural network‐based inverse design scheme was implemented using PyTorch on a PyCharm platform with Python, running on a workstation equipped with an Intel Xeon Gold 5218 CPU (2.3 GHz), 128 GB RAM, and an RTX 2080 Ti GPU. During the optimization, the Adam optimizer^[^ ^47^ ^]^ with a learning rate of 0.001 was employed, and Gaussian noise (uniformly distributed between 0 and 0.05) was added to input patterns at each iteration to enhance convergence. After the network optimization, the resultant mapping function $R_{\theta^{*}}$ can then be used to reconstruct the phase profiles as follows:

(3) $$\left(\Delta \tilde{\phi_{A}}\left(x,y\right),\Delta \tilde{\phi_{B}}\left(x,y\right)\right)=R_{\theta^{*}}\left(I_{i}\right)$$

where $\Delta \tilde{\phi_{A}}\left(x,y\right)$ and $\Delta \tilde{\phi_{B}}\left(x,y\right)$ denote the optimized phase profiles.

According to the above formulations, the quality of the reconstructed acoustic intensity fields is highly dependent on the distance and in‐plane rotation angle between two cascaded AHLs. To determine the optimal value ranges of these two parameters, their effects on the quality of reconstructed acoustic intensity fields were first quantitatively investigated (Section S2; Figure S1a,b, Supporting Information). The peak signal‐to‐noise ratio (PSNR) was employed to evaluate reconstruction quality, where a higher PSNR typically indicates better reconstruction accuracy. Through this approach, our proposed physics‐driven neural network‐based inverse design scheme can effectively optimize the phase profiles of two cascaded AHLs, thereby enabling high‐fidelity multi‐dimensional acoustic encryption and high‐quality visual decryption.

### 2.3 Experimental Demonstrations

#### 2.3.1 1D Acoustic Encryption and Visual Decryption

**Figure 3:**
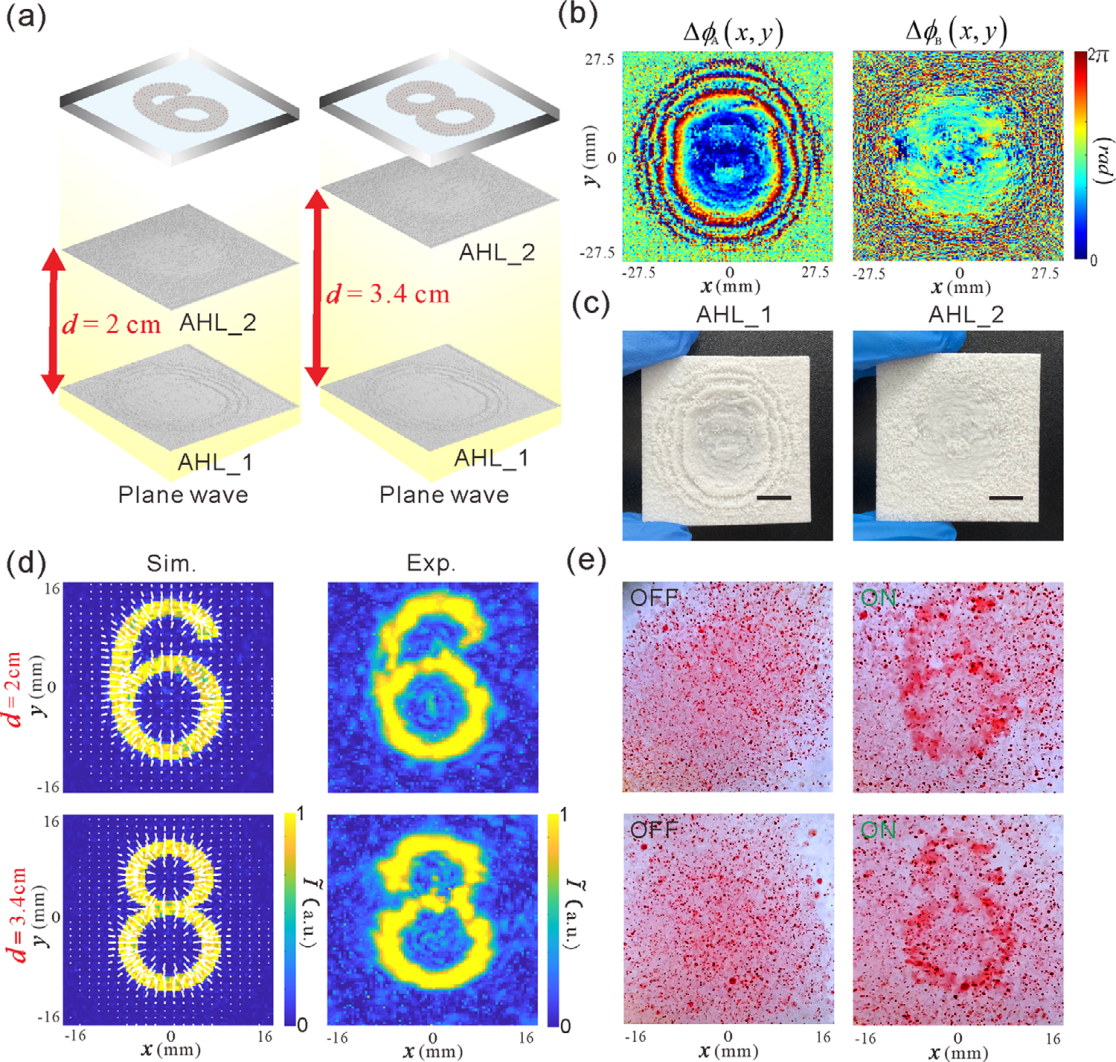
a) Design principle and schematics for 1D acoustic encryption and visual decryption via manipulating colored PDMS microparticles. Here, beyond phase profiles, the distance *d* between two cascaded AHLs is used as an additional secret key to encode encrypted holographic images of the digits “6” and “8”, realizing 1D acoustic encryption and visual decryption. b) Optimized phase profiles of two AHLs. c) Photograph of the 3D‐printed samples of two AHLs. The scale bars in (c) are 110 µm. d) Simulated and experimental results of encrypted holographic images (acoustic intensity fields), where the length and direction of the white arrows superimposed on the simulated results indicate the relative magnitude and direction of ARFs exerted on the PDMS microparticles, respectively. e) Experimental results of the visual decryption dynamics of the colored PDMS microparticles when the acoustic power is off(left)and on (right).

**Figure** **3**a illustrates the design and concept of 1D acoustic encryption and visual decryption based on our VMD‐CHAE device. When AHL_2 is moved away from AHL_1 along the diffractive path by a design distance *d* = 2cm, the design encrypted holographic image of the digit “6” is visualized at the image plane *z_t_* = 5cm within the acoustic window. When the distance between two cascaded AHLs is adjusted to *d* = 3.4cm, another design encrypted holographic image of the digit “8” is decrypted and visualized at the image plane *z_t_* = 5cm within the acoustic window. In this way, multiple design encrypted holographic images are encoded onto different distances between two cascaded AHLs, realizing 1D acoustic encryption and visual decryption. Figure 3b presents the calculated phase profiles of two AHLs, while the 3D‐printed samples of two AHLs (see Experimental Section for details) are shown in Figure 3c. The 3D‐printed samples have a size of 550 × 550µm^2^ and contain 160 × 160 pixels. The simulated and measured acoustic intensity fields recorded over the image plane are shown in Figure 3d. As observed in Figure 3d, although the experimental measurements are slightly less uniform than the simulated ones, the shape of the measured acoustic intensity fields remains very close to the design, highlighting the favorable acoustic encryption performance of the VMD‐CHAE device. More importantly, the measured acoustic intensity fields exhibit strong energy focusing at the positions of the encrypted holographic images, which will contribute to the precision and robustness of particle manipulation for visualizing the designed encrypted holographic images. To verify the holographic visual decryption performance of the VMD‐CHAE device, experiments were conducted using the colored PDMS microparticles. Figure 3e and Movie S1 (Supporting Information) illustrate the visual decryption dynamics of the colored PDMS microparticles when the distances between two cascaded AHLs are *d* = 2cm and *d* = 3.4cm, respectively. Initially, the PDMS microparticles are randomly distributed and suspended on the water surface within the acoustic window, as shown in Figure 3e. When the ultrasonic power is turned on, ARFs propel the PDMS microparticles toward regions of high acoustic field intensity (Figure 3d). Consequently, after 5 s, the PDMS microparticles rapidly assemble into the shape of the encrypted holographic image of the digit “6”. Similarly, when the distance is adjusted to *d* = 3.4cm, the PDMS microparticles are redistributed to form a particle pattern matching the encrypted holographic image of the digit “8” within 14 s, as illustrated in Figure 3e and Movie S1 (Supporting Information). To further test the security of 1D acoustic encryption, we investigated the sensitivity of the deviation of distance between two cascaded AHLs. Figure S2 (Section S3, Supporting Information) presents some reconstructed acoustic intensity fields under different deviated distances between two cascaded AHLs. As shown in Figure S2 (Supporting Information), both reconstructed encrypted images become blurred when the deviation from the designed distance between two cascaded AHLs exceeds 3λ. Therefore, in addition to phase profiles, the distance between two cascaded AHLs provides an extra DOF to enhance the information security. These results demonstrate that our VMD‐CHAE device can not only achieve high‐security 1D acoustic encryption by adjusting the distance between two cascaded AHLs, but also enable rapid holographic visual decryption.

#### 2.3.2 2D Acoustic Encryption and Visual Decryption

**Figure 4:**
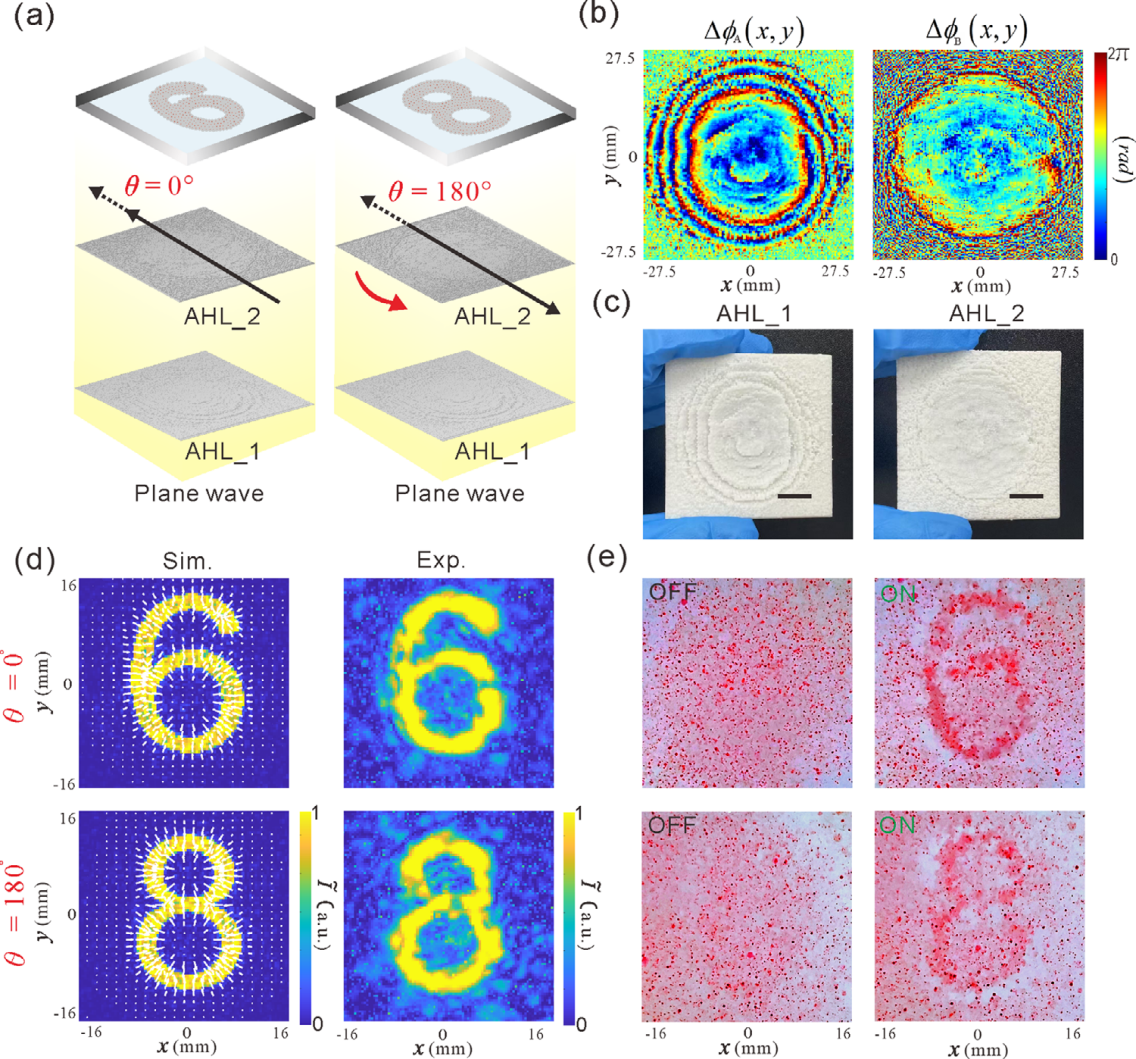
a) Design principle and schematics for 2D acoustic encryption and visual decryption via manipulating colored PDMS microparticles. Here, beyond phase profiles, the in‐plane rotation angle θ between two cascaded AHLs is used as an additional secret key to encode encrypted holographic images of the digits “6” and “8”, realizing 2D acoustic encryption and visual decryption. b) Optimized phase profiles of two AHLs. c) Photograph of the 3D‐printed samples of two AHLs. The scale bars in (c) are 110 µm. d) Simulated and experimental results of encrypted holographic images (acoustic intensity fields), where the length and direction of the white arrows superimposed on the simulated results indicate the relative magnitude and direction of ARFs exerted on the PDMS microparticles, respectively. e) Experimental results of the visual decryption dynamics of the colored PDMS microparticles when the acoustic power is off(left)and on (right).

In the following, we further investigate the feasibility and performance of 2D acoustic encryption and visual decryption based on our VMD‐CHAE device. As illustrated in **Figure** **4**a, the distance between two cascaded AHLs is fixed at *d* = 2cm. When AHL_2 is rotated counterclockwise by two distinct in‐plane rotation angles θ = 0° and θ = 180°, two independent holographic images of the digits “6” and “8” can be decrypted and visualized at the image plane *z_t_* = 5 cm within the acoustic window, respectively. In other words, multiple design holographic images can be encrypted by rotating one of the cascaded AHLs around the normal at its geometric center, realizing 2D acoustic encryption and visual decryption. The optimized phase profiles of two AHLs and their fabricated samples are shown in Figure 4b,c respectively. Figure 4d presents the corresponding simulated and experimental results of the reconstructed acoustic intensity fields. As shown in Figure 4d, the experimentally reconstructed acoustic intensity fields exhibit slight distortions and interference artifacts. Nevertheless, they still retain recognizable features of two encrypted holographic images, with overall satisfactory reconstruction quality. Furthermore, in the experimental measurements, most of the acoustic energy is intensely focused on the target pixels of two encrypted holographic images, which facilitates clear visual decryption of the encrypted information through particle manipulation. To testify the holographic visual decryption performance of the VMD‐CHAE device for 2D acoustic encryption, experiments of manipulation the colored PDMS microparticles were carried out. As depicted in Figure 4e and Movie S2 (Supporting Information), when the ultrasonic power is activated and AHL_2 is rotated counterclockwise by a design in‐plane rotation angle of θ = 0°, the randomly suspended PDMS microparticles gradually migrate toward regions of high acoustic intensity due to ARFs, forming a particle pattern corresponding to the encrypted digit “6” within 5 s in the acoustic window. Similarly, when AHL_2 is rotated counterclockwise by another design in‐plane rotation angle of θ = 180°, the PDMS microparticles rapidly reorganize to form a particle pattern matching the design encrypted holographic image of the digit “8” within the acoustic window, as described in Figure 4e and Movie S2 (Supporting Information). To further examine the security of 2D acoustic encryption, we investigated the impact of the deviation of in‐plane rotation angle between two cascaded AHLs. Figure S3 (Section S3, Supporting Information) shows some reconstructed acoustic intensity fields under different deviated in‐plane rotation angles between two cascaded AHLs. As shown in Figure S3 (Supporting Information), even with an in‐plane rotation angle deviation as small as 6° between two cascaded AHLs, the diffraction efficiency and uniformity of two reconstructed encrypted images deteriorate seriously. Hence, the in‐plane rotation angle between two cascaded AHLs can effectively serve as an additional secret key, which, combined with the phase profiles, enhances information security. These results confirm that our VMD‐CHAE device can achieve high‐security 2D acoustic encryption by modulating the in‐plane rotation angle of two cascaded AHLs, while providing intuitive visual decryption via rapid particle manipulation.

#### 2.3.3 3D Acoustic Encryption and Visual Decryption

**Figure 5:**
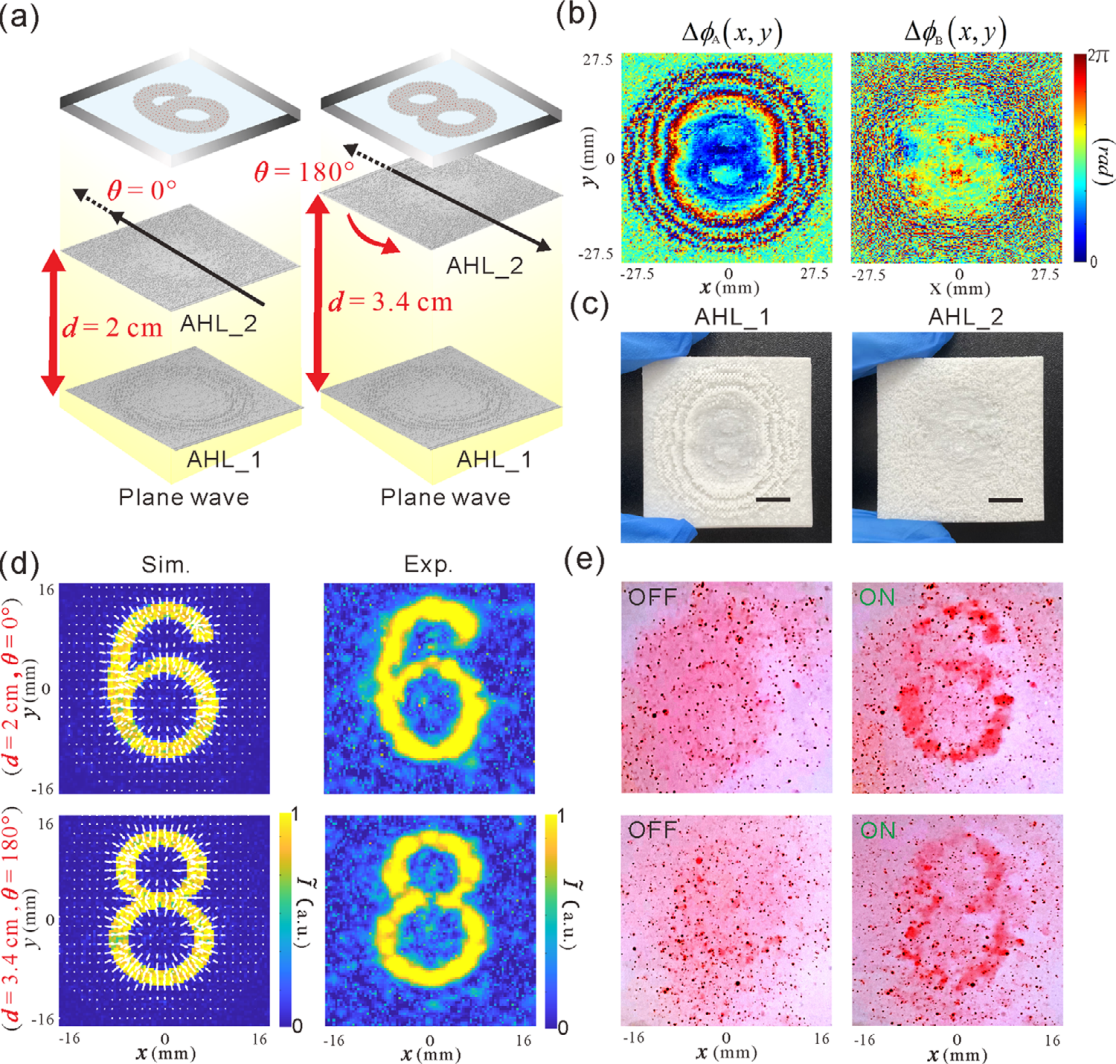
a) Design principle and schematics for 3D acoustic encryption and visual decryption via manipulating colored PDMS microparticles. Here, beyond phase profiles, both the distance *d* and in‐plane rotation angle θ between two cascaded AHLs are used as additional secret keys to encode encrypted holographic images of the digits “6” and “8”, realizing 3D acoustic encryption and visual decryption. b) Optimized phase profiles of two AHLs. c) Photograph of the 3D‐printed samples of two AHLs. The scale bars in (c) are 110 µm. d) Simulated and experimental results of encrypted holographic images (acoustic intensity fields), where the length and direction of the white arrows superimposed on the simulated results indicate the relative magnitude and direction of ARFs exerted on the PDMS microparticles, respectively. e) Experimental results of the visual decryption dynamics of the colored PDMS microparticles when the acoustic power is off(left)and on (right).

We further demonstrate that acoustic encryption and visual decryption based on our VMD‐CHAE device can be extended to three dimensions to enhance information security. The design and concept of 3D acoustic encryption and visual decryption based on our VMD‐CHAE device are shown in **Figure** **5**a, where both the distance and in‐plane rotation angle of the AHL_2 are modulated simultaneously. Specifically, when the AHL_2 is moved away from AHL_1 by a design distance *d* = 2cm and rotated counterclockwise by a design in‐plane rotation angle θ = 0°, the design encrypted holographic image of the digit “6” is visualized at the image plane *z_t_* = 5cm within the acoustic window. By simultaneously adjusting the distance and in‐plane rotation angle of the AHL_2 relative to the AHL_1 to the new values (*d* = 3.4cm, θ = 180°), the design encrypted holographic image of the digit “8” is decrypted and visualized at the image plane *z_t_* = 5cm within the acoustic window. As a result, multiple design holographic images can be encrypted by simultaneously varying the distance and in‐plane rotation angle, achieving 3D acoustic encryption and visual decryption. Figure 5b,c provides the calculated phase profiles of two AHLs and corresponding fabricated samples, respectively. The simulated and experimental acoustic intensity fields of 3D acoustic encryption are displayed in Figure 5d. A simple visual comparison and analysis of Figures 3, 4, and 5d reveal that the experimental acoustic intensity fields of 3D acoustic encryption exhibit higher magnitudes and greater fidelity across two encrypted images than those from 1, and 2D acoustic encryption demonstrations. This enhancement in the experimental acoustic intensity fields arises from the increased DOFs in 3D encoding, which generate larger and more uniform ARFs, thereby facilitating more precise and robust particle patterning for visualizing encrypted holographic images. To validate the enhanced visual decryption performance of the VMD‐CHAE device, experiments were performed to manipulate the colored PDMS microparticles. Movie S3 (Supporting Information) displays the visual decryption dynamics of manipulating the colored PDMS microparticles when the distance and in‐plane rotation angle of the AHL_2 are sequentially set as (*d* = 2cm, θ = 0°) and (*d* = 3.4cm, θ = 180°). As expected, compared with the visual decryption dynamics observed in the 1D and 2D acoustic encryption demonstrations (Movies S1 and S2, Supporting Information), the PDMS microparticles exhibit enhanced directed movement toward the encrypted patterns, which results in higher‐quality particle patterns with improved spatial morphology and reduced formation time. To analyze the crack difficulty of 3D acoustic encryption, a series of numerical simulations were conducted, in which certain deviations were simultaneously introduced to the designed distances and in‐plane rotation angles (secret keys) between two cascaded AHLs. For an intuitive demonstration, Movie S4 (Supporting Information) shows the dynamic evolution process of the reconstructed encrypted images as these two secret keys are gradually deciphered. Some intermediate results from Movie S4 (Supporting Information) are presented in Figure S4 (Section S3, Supporting Information). As revealed in Movie S4 and Figure S4 (Supporting Information), even if one of the secret keys combined with the phase profiles is stolen, a small deviation in the other secret key will cause severe degradation in the quality of two encrypted images. Two encrypted images can be precisely decrypted, only when both secret keys, combined with the phase profiles, are correct. To further demonstrate the flexibility and general applicability of the device, we also numerically and experimentally demonstrate the high‐quality 3D acoustic encryption and rapid visual decryption for letters (“C” and “D”) (Section S4; Figure S5 and Movie S5, Supporting Information). These results demonstrate that our VMD‐CHAE device exhibits enhanced encryption security and high‐quality visual decryption capabilities in 3D acoustic encryption.

### 2.4 Performance Analysis and Discussion

**Figure 6:**
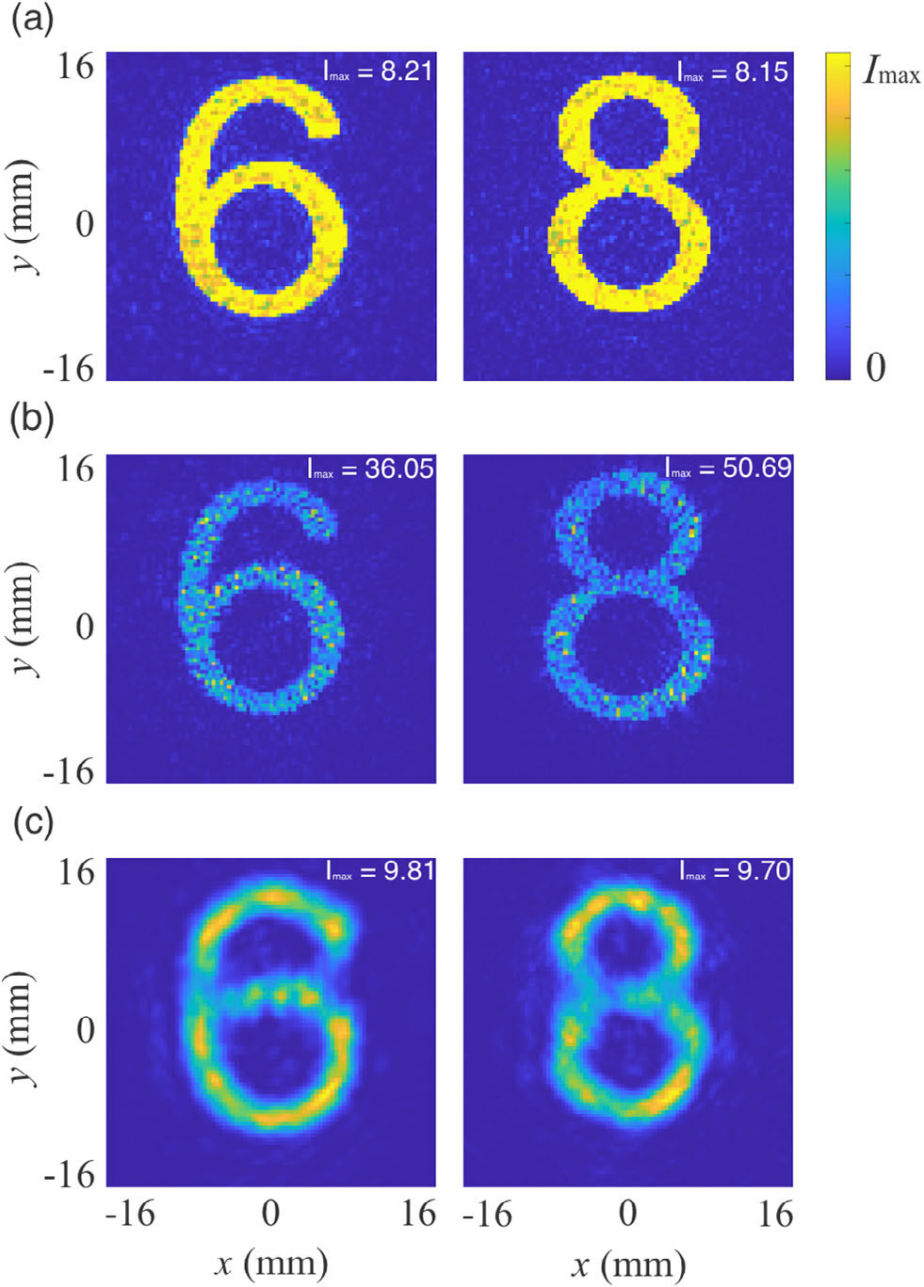
Reconstructed acoustic intensity fields of the digits “6” and “8” with the proposed physics‐driven scheme (a), modified IASA (b), and conventional data‐driven scheme (c). I_max _ indicates the maximum intensity of the reconstructed acoustic intensity fields of the target patterns.

To investigate the influence of the optimization methods on the performance of the device, we first compare the proposed physics‐driven neural network‐based inverse design scheme with the modified iterative angular spectrum approach (IASA)^[^ ^48^ ^]^ and conventional data‐driven scheme^[^ ^41^ ^]^ (Detailed implementation, parameter setting, and quantitative comparison see Section S5 and Table S1, Supporting Information). It is clearly observed from **Figure** **6**a,b that the reconstructed intensity fields of the proposed scheme show uniform intensity over the patterns with minimal artifacts compared with those of modified IASA, although the reconstructed intensity fields of modified IASA have higher diffraction efficiency. As a result, the corresponding average PSNR of the proposed scheme are 21.31 dB, which is much better than that of the modified IASA (15.99 dB). This is attributed to the fact that the modified IASA is optimized solely through iterative forward and backward wave propagation, lacking an explicit optimization process tailored to reconstruction accuracy. The conventional data‐driven scheme employ the same network structure, physical models and loss function, but it is trained to fit the training data set (12, 000 handwritten digits from the Modified National Institute of Standards and Technology (MNIST) database^[^ ^49^ ^]^) in an unsupervised learning manner. After training, we fed the trained network with the digits “6” and “8” that are different from the MNIST database. Figure 6c and Table S1 (Section S5, Supporting Information) show that the trained network can quickly and successfully predict cascaded holograms for these unseen target patterns within 0.05s. However, a simple visual inspection of Figure 6a,c indicates that the reconstructed intensity fields with conventional data‐driven scheme show much lower magnitude and larger variation in intensity over the patterns than those with the proposed physics‐driven scheme. Quantitatively, the average PSNR of the conventional data‐driven scheme is 18.79 dB, which is about 2.52 dB lower than that of the proposed physics‐driven scheme. This is plausible because the conventional data‐driven scheme learns a mapping function from the statistics of a large training dataset, such that reconstruction quality reasonably degrades when target patterns deviate from the training samples. By contrast, the reconstruction quality of the proposed physics‐driven scheme is not similarly compromised, as such scheme is optimized through the interplay between network structure and the physical model. The convergence process and selected intermediate reconstructed intensity fields of the proposed physics‐driven scheme presented in Figures S6 and S7 (Section S6, Supporting Information) further confirm that the fields gradually converge to the target patterns as the number of iterations increases. Note that the computation speed of the proposed physics‐driven scheme is much slower than that of existing methods (the modified IASA and conventional data‐driven scheme), which can be improved by extending the loss function with additional terms (such as diffraction efficiency loss or phase variation loss)^[^ ^50^ ^]^ or by adopting a co‐optimization strategy that integrates both data‐driven and physics‐driven schemes.^[^ ^51^ ^]^ These results demonstrate that the proposed physics‐driven scheme achieves higher‐quality reconstructed intensity fields than existing methods, thereby facilitating higher‐fidelity encryption and higher‐quality visual decryption of encrypted information via particle manipulation.

In addition to optimization methods, the number of encrypted images also exerts a significant influence on device performance. Figure S8a (Section S7, Supporting Information) presents the variation of average PSNR as a function of the number of encrypted images (digits and letters). Specifically, an increasing number of encrypted images leads to longer computation time (Section S7, Figure S8b, Supporting Information) and a dramatic drop in average PSNR. This indicates gradual degradation in the quality of reconstructed acoustic intensity fields and further compromises particle manipulation precision and robustness. Additionally, the average PSNR values for letters are considerably lower than those for digits, suggesting that the complexity of encrypted images is also a key factor. Apart from the aforementioned design parameters, fabrication or assembly errors in practical manufacturing and experimental setups (e.g., misalignment or slight tilt) may exert a notable impact on device performance. Figure S9a (Section S8, Supporting Information) depicts the variation of average PSNR as a function of misalignment between two AHLs along the x‐direction. When the misalignment is below 0.2 λ (≈ 0.15 mm), the average PSNR remains at 21.31 dB with no loss in reconstruction quality. Figure S9b (Section S8, Supporting Information) illustrates the variation of average PSNR versus the tilt angle of AHL_2 along the z‐direction. Even when the tilt angle reaches 7°, the average PSNR only decreases by 2 dB. Since these manufacturing tolerances fall within the precision range of our 3D printer, our VMD‐CHAE device can achieve high‐fidelity encryption and superior visual decryption in experiments.

Compared to existing acoustic encryption methods, our VMD‐CHAE device offers several distinct advantages. First, beyond the phase profiles, the encrypted images are concealed in the combination of the distance and in‐plane rotation angle between the two cascaded AHLs. Even if the two AHLs are stolen and cascaded with the correct distance, the encrypted images cannot be precisely decrypted without the accurate in‐plane rotation angle between them. This imposes more stringent conditions for decryption and minimizes the risk of information leakage, thereby significantly enhancing encryption security. Moreover, the multi‐dimensional multiplexing technique increases the DOFs for encoding the encrypted images, which facilitates improved reconstruction quality of the experimental acoustic intensity fields. Notably, this distinctive advantage enables rapid holographic visual decryption of encrypted information through particle manipulation. This not only greatly boosts decryption efficiency but also eliminates the need for complex auxiliary instrumentation or intricate signal processing to reveal the encrypted information, which has not yet been realized by existing acoustic encryption techniques.

## 3 Conclusions

In summary, we have integrated the concept of multi‐dimensional multiplexing cascaded acoustic holography with particle manipulation to develop a compact acoustic encryption device that simultaneously enables multi‐dimensional information encryption and provides immediate visual holographic decryption for the first time. Unlike existing encryption schemes based on acoustic holograms, our VMD‐CHAE device further utilizes the distance and in‐plane rotation angle between two AHLs, in addition to the phase profiles, as two additional DOFs for multi‐dimensional information encryption, which significantly enhances the design versatility and information security. More importantly, our VMD‐CHAE device uniquely provides intuitive and informative decryption of secret information without the need for additional complex mechanical or electronic components, thus rendering it potentially compatible with micro‐electromechanical systems (MEMS) and wearable systems. The practicality and feasibility of the VMD‐CHAE device are validated through one‐, two‐, and 3D acoustic encryption experiments, demonstrating enhanced security encryption and high‐quality instantaneous visual decryption. While our proof‐of‐concept results demonstrate the feasibility and superiority of our VMD‐CHAE device, further optimizations are warranted to enhance its performance, which can be pursued through multidimensional strategies. Computationally, the proposed physics‐driven scheme could be refined by expanding the loss function with metrics like diffraction efficiency loss or phase variation loss,^[^ ^50^ ^]^ or adopting a hybrid co‐optimization paradigm integrating data‐driven and physics‐driven approaches.^[^ ^51^ ^]^ Mechanically, the simple two‐layer stands could be replaced with high‐precision 3D translation stages to enable more accurate alignment, distance control, and in‐plane rotation of the two AHLs, which facilitates the design and fabrication of AHLs with higher pixel resolution to boost encrypted image information capacity and complexity. Architecturally, cascaded AHLs could be redesigned using novel methodologies (e.g., ultrathin metasurfaces^[^ ^52^ ^]^ or high‐resolution manifold acoustic holography^[^ ^26^ ^]^) and subsequently fabricated via emerging fabrication approaches (e.g., picosecond ultraviolet laser processing,^[^ ^26^ ^]^ projection micro stereolithography system,^[^ ^32^ ^]^ or silicon microfabrication techniques^[^ ^52^ ^]^) to reduce device size while increasing pixel resolution, thereby advancing potential integration with MEMS and wearable platforms.

To the best of our knowledge, this is the first experimental demonstration of a multi‐dimensional acoustic cascaded holographic encryption device with instantaneous visual decryption via particle manipulation. It greatly advances the practicality and feasibility of current acoustic hologram‐based encryption schemes for real‐world applications. We believe that our VMD‐CHAE device offers significant advantages over conventional acoustic encryption schemes based on acoustic holograms, including its simple architectural design, compact size, relieving the burden of time‐consuming mechanical scanning and complex signal processing, clear and instantaneous visual decryption, and enhanced security. These features make it highly promising for a wide range of applications, such as acoustic information storage and processing, underwater communications, volumetric displays, dynamic particle manipulation, tissue engineering, and medical ultrasound.

## 4 Experimental Section

### Fabrication of Acoustic Holographic Lens

The AHL samples were fabricated using a Low Force Stereolithography 3D printer (Formlabs, Form 3+), with white resin chosen as the holographic material. The sound speed of the resin was measured to be 2538 m s^−1^ at 2 MHz. The detailed fabrication process is as follows:

1. Phase Profile Optimization: Obtain the optimized phase profiles of two AHLs $\Delta \tilde{\phi_{A}}\left(x,y\right)$ and $\Delta \tilde{\phi_{B}}\left(x,y\right)$ using the proposed physics‐driven neural network‐based inverse design scheme.
2. Thickness Profile Conversion: Convert the optimized phase profiles of two AHLs into thickness profiles *T_A_* (*x*,*y*) and *T_B_* (*x*,*y*) using following Equations (4) and (5):
(4) $$\Delta {\tilde{\phi }}_{A/B}\left(x,y\right)=\left(k_{L}-k_{W}\right)T_{A/B}\left(x,y\right)$$
(5) $$T_{A/B}\left(x,y\right)=T_{0}-T_{A/B}\left(x,y\right)$$
  where *k_L_* and *k_W_* denote the wavenumbers of the AHLs and background medium, respectively, (*x*, *y*) denotes the coordinates of each hologram pixel. *T*_0_ is the initial thickness of the AHLs, which was set to 1.5 mm to ensure structural robustness.
3. 3D Printing: Export thickness profiles *T_A_* (*x*,*y*) and *T_B_* (*x*,*y*)in Standard Tessellation Language (STL) format, then load these two print STL files into the Low Force Stereolithography 3D printer for printing.

After the above processes, the AHL samples were fabricated with a pixel dimension of 550 × 550 µm^2^ and a pixel resolution of 160 × 160, as shown in Figures 3, 4, and 5c, respectively.

### Experimental Setup for Pressure Fields Measurement

As schematically illustrated in Figure S10a (Section S9, Supporting Information), acoustic pressure field measurements were conducted in a water tank with dimensions of ≈500 × 600 × 1000 mm^3^. To eliminate the influence of reflected waves, a series of sinusoidal electrical signals were generated using a multi‐function waveform generator (RIGOL DG4202, China). These signals were converted into ultrasonic waves by a custom transducer with a central frequency of 2 MHz, driven by a 5‐cycle sine wave with a peak voltage of 10 V at 2 MHz. The acoustic waves were modulated by two cascaded AHLs and propagated to the target plane. The resultant pressure fields were scanned using a pin hydrophone (NH0500, 500 µm diameter) mounted on a three‐axis computer‐controlled positioning system (Precision Acoustics UMS3), with a spatial step size of 300 µm and 120 steps in each x/y dimension. Data were recorded via a digital oscilloscope (Agilent DSO‐X‐3034A) and processed using Precision Acoustics software. To enable 1D, 2D, and 3D acoustic encryption, two 3D‐printed two‐layer stands were fabricated. As shown in Figure S10b (Section S9, Supporting Information), the interlayer spacings of two stands were set to 2 and 3.4 cm to provide two distinct distances. Each stand features a central square hole (550 × 550 µm^2^) to maintain lateral alignment and permit two in‐plane rotation angles of 0° and 180° between the AHLs. This modular design facilitates the flexible implementation of multi‐dimensional acoustic encryption experiments.

### Calculation and Analysis of ARFs

Based on Gor'kov theory,^[^ ^45^ ^]^ the acoustic trapping of particles is predominantly governed by the gradient force derived from the acoustic field. In this work, the colored PDMS microparticles fall within the Rayleigh regime, with a radius < 60 µm, which is far smaller than the wavelength of the incident acoustic wave (750 µm). Consequently, the ARFs acting on these particles can be quantified using the following Equations (6) and (7):^[^ ^43^ ^]^

(6) $$F_{rad}=A\Phi \left(\tilde{\kappa },\tilde{\rho }\right)kr^{3}\sin\left(2kx\right)$$

(7) $$\Phi \left(\tilde{\kappa },\tilde{\rho }\right)=\frac{1}{3}\left(\frac{5\tilde{\rho }-2}{2\tilde{\rho }+1}-\tilde{\kappa }\right)$$

where *F_rad_* denotes the ARFs exerted on particles, *A* is a constant, $\Phi (\tilde{\kappa },\tilde{\rho })$ represents the contrast factor that is a function of the relative density $\tilde{\rho }=\rho_{p}/\rho_{0}$ and relative compressibility $\tilde{\kappa }=\kappa_{p}/\kappa_{0}$ between the particles and the surrounding medium, κ_*p*_, κ_0_, ρ_*p*_, and ρ_0_are the compressibility and density of particles and the surrounding medium, respectively.

According to the above ARFs calculation formulas, the acoustic contrast factor $\Phi (\tilde{\kappa },\tilde{\rho })$ determines the directionality of the ARFs. Particles with a positive ​acoustic contrast factor (i.e., higher density and sound speed than the medium) migrate toward pressure nodes (regions of low acoustic field intensity), while those with a negative acoustic contrast factor move toward pressure anti‐nodes (regions of high acoustic field intensity).^[^ ^44^ ^]^ In this work, the colored PDMS microparticles exhibit a negative acoustic contrast factor, causing them to be trapped at regions of high acoustic field intensity due to the ARFs. As a result, the PDMS microparticles assemble into the shape of the encrypted holographic images.

### Experimental Setup for Particle Manipulation

As schematically depicted in Figure S11a (Section S9, Supporting Information), the particle manipulation experiments were conducted in an acoustic window submerged in a water tank. The acoustic window was made of PDMS, with a wall thickness of ≈3 mm (≈ 4λ) to absorb stray acoustic waves and minimize reflections. Additionally, the bottom of the acoustic window was sealed with an 80 µm (≈ 0.1λ)‐thick PDMS membrane, which further reduces reflections while allowing acoustic waves to propagate into the window with minimal interference. Before the manipulation, the colored PDMS microparticles were randomly distributed and suspended on the water surface within the acoustic window using a transfer pipette. The water surface was aligned with the image plane using an auxiliary frame to ensure precise positioning. During the manipulation, a stable, high‐power amplifier (Mini‐Circuits, LZY‐22+, USA) was connected to the homemade transducer, delivering a peak voltage of approximately 400 mV to provide sufficient energy for particle manipulation. When the fabricated AHLs were placed on a 3D‐printed two‐layer stand with a design distance or/and in‐plane rotation angle, the ARFs propelled the colored PDMS microparticles toward regions of high acoustic intensity. This resulted in the formation of a particle pattern that precisely aligned with the location of the encrypted holographic image. The dynamics of the entire particle manipulation process were captured using a camera mounted above the water surface.

## Conflict of Interest

The authors declare no conflict of interest.

## Supporting information



Supporting Information

Supplemental Movie 1

Supplemental Movie 2

Supplemental Movie 3

Supplemental Movie 4

Supplemental Movie 5

## Data Availability

The data that support the findings of this study are available from the corresponding author upon reasonable request.
