# Supplementary material for: Multi‐Dimensional Acoustic Cascaded Holographic Encryption with Instantaneous Visual Decryption via Particle Manipulation
Source: Adv Sci (Weinh). 2025 Dec 19;13(12):e16151. doi: 10.1002/advs.202516151 (PMC12948201; doi:10.1002/advs.202516151)
Supplement: Supplementary file 1 — Supporting Information [file ADVS-13-e16151-s003.docx]

Supporting Information

Multi-dimensional Acoustic Cascaded Holographic Encryption with Instantaneous Visual Decryption via Particle Manipulation

Qin Lin, Feiyan Cai*, Yunqing Liu, Zejiao Zhou, Jiayang Li, Rujun Zhang, Hongpeng Chen, Hairong Zheng*, and Huailing Zhang*

1. Lin, Y. Liu, Z. Zhou, J. Li, H. Chen, H. Zhang

School of Biomedical Engineering

Dongguan Key Laboratory of Medical Electronics and Medical Imaging Equipment

Songshan Lake Innovation Center of Medicine & Engineering

Guangdong Medical University

Dongguan 523808, P. R. China

E-mail: huailing@163.com

1. Cai, R. Zhang, H. Zheng

Paul C. Lauterbur Research Center for Biomedical Imaging

Shenzhen Institutes of Advanced Technology

The Key Laboratory of Biomedical Imaging Science and System

Chinese Academy of Sciences

Shenzhen 518055, P. R. China

E-mail: [fy.cai@siat.ac.cn;](mailto:fy.cai@siat.ac.cn;) hr.zheng@siat.ac.cn

**This file includes:**

Section S1: Theory of wave propagation through cascaded AHLs

Section S2: The effects of the distance and in-plane rotation angle between two cascaded AHLs on the quality of reconstructed acoustic intensity fields

Section S3: Vulnerability tests of the one-, two-, and three-dimensional acoustic encryption

Section S4: Example of three-dimensional acoustic encryption and visual decryption test for letters

Section S5: Performance comparison for different optimization methods

Section S6: The convergence process of the proposed scheme for the three-dimensional acoustic encryption

Section S7: The effects of the number and complexity of encrypted images on the device's performance

Section S8: The effects of the misalignment and tilt angle between two cascaded AHLs on the device's performance

Section S9: Experimental setups for acoustic fields measurement and particle manipulation

Figures S1-S9

Table S1

Movie S1: Visual decryption dynamics of one-dimensional acoustic encryption

Movie S2: Visual decryption dynamics of two-dimensional acoustic encryption

Movie S3: Visual decryption dynamics of three-dimensional acoustic encryption

Movie S4: Dynamic evolution process of deciphering encrypted images

Movie S5: Visual decryption dynamics of three-dimensional acoustic encryption for letters

**Section S1: Theory of wave propagation through cascaded AHLs**

When two cascaded AHLs are considered as infinitely thin, the propagation behavior of acoustic waves through them can be analytically modeled using angular spectrum diffraction theory, as established in prior studies.^[1-6]^ Specifically, as illustrated in Figure 2, for the *i*^th^ cascaded configuration, AHL_1 with phase profile is assumed to be located at the $z_{1}=0$cm plane, while AHL_2 with phase profile is positioned at a design distance $d=d_{i}$ from AHL_1 and rotated by a design in-plane rotation angle $\theta=\theta_{i}$. When the incident plane acoustic waves pass through AHL_1 and reach the plane $z_{2}^{i}$ immediately before AHL_2, the resultant complex acoustic field is given by:

$u_{2}^{in}\left(x,y,z_{2}^{i}\right)=F^{-1}\left\{F\left[u_{1}\left(x,y,z_{1}\right)\right]\cdot H\left(k_{x},k_{y},d_{i}\right)\right\}$ （S1）

（S2）

$H\left(k_{x},k_{y},d_{i}\right)=e^{-jd_{i}\sqrt{k^{2}-k_{x}^{2}-k_{y}^{2}}}$ （S3）

where $F$ and $F^{-1}$ denote the Fourier transform and inverse Fourier transform, respectively. $u_{1}\left(x,y,z_{1}\right)$represents the complex acoustic field immediately after transmission through AHL_1. $U_{0}\left(x,y\right)$ is the pressure amplitude of the incident acoustic waves. $H\left(k_{x},k_{y},d_{i}\right)$ denotes the spectral propagator for $k_{x}^{2}+k_{y}^{2}\leq k^{2}$, where $k_{x}$ and $k_{y}$ are spatial frequencies, and $k$ is the wavenumber of the surrounding medium. $d_{i}=z_{2}^{i}-z_{1}$ is the *i*^th^ design distance between two AHLs.

Eq. (S1) can be reformulated as:

（S4）

where $U_{2}^{in}\left(x,y,z_{2}^{i}\right)$ and denote the amplitude and phase profiles of $u_{2}^{in}\left(x,y,z_{2}^{i}\right)$, respectively. Subsequently, the acoustic waves propagate through the rotated AHL_2. Since the rotated AHL_2 is rotated by a design in-plane rotation angle $\theta_{i}$ relative to the original phase profile , it acquires a new phase profile, denoted as . Thus, the modulated acoustic field immediately after transmission through the rotated AHL_2 can be expressed as:

（S5）

Consequently, the acoustic waves enter the acoustic window and reach the image plane $z_{t}$. The resultant acoustic intensity field can be calculated by:

（S6）

$u_{t}^{i}\left(x,y,z_{t}\right)=F^{-1}\left\{F\left[u_{2}^{out}\left(x,y,z_{2}^{i}\right)\right]\cdot H\left(k_{x},k_{y},z_{t}-z_{2}^{i}\right)\right\}$ （S7）

where $u_{t}^{i}\left(x,y,z_{t}\right)$ represents the complex acoustic field at the image plane $z_{t}$. denotes the forward propagation function that maps the phase profiles and of two AHLs to the acoustic intensity field $\tilde{I}_{i}\left(x,y,z_{t}\right)$ for a given combination of distance $d=d_{i}$ and in-plane rotation angle $\theta=\theta_{i}$.

**Section 2: The effects of the distance and in-plane rotation angle between two cascaded AHLs on the quality of reconstructed acoustic intensity fields**

To first investigate the effect of the distance between two cascaded AHLs, a set of acoustic holograms were calculated: each case was designed to generate two encrypted holographic images (digits “6” and “8”) at the image plane $z_{t}=5$cm by moving AHL_2 to two distinct distances away from AHL_1, respectively. The first distance was set to $d=2$cm, while the second distance was then varied from 2.2 cm to 4.8 cm. The resulting variation of average PSNR of two encrypted holographic images for each case as a function of the second distance can be seen in Figure S1a. From Figure S1a, it can be observed that the average PSNR fluctuates slightly, with most values exceeding 20 dB. Considering both the average PSNR and experimental feasibility, the optimal second distance was determined to be $d=3.4$cm. To further test the effect of the in-plane rotation angle between two cascaded AHLs, a set of acoustic holograms were calculated to generate two encrypted holographic images (digits “6” and “8”) at the image plane $z_{t}=5$cm by rotating AHL_2 counterclockwise to two distinct in-plane rotation angles relative to AHL_1, respectively. The distance between two cascaded AHLs was fixed at $d=2$cm. The first in-plane relative rotation angle was set to $\theta=0^{\circ}$, while the second in-plane relative rotation angle was increased from 45° to 315°. The resulting variation of the average PSNR of two encrypted holographic images for each case as a function of the second in-plane relative rotation angle is displayed in Figure S1b. As shown in Figure S1b, the average PSNR first increases and then decreases, reaching a maximum value of 21.31 dB at 180°. As a result, the optimal second in-plane relative rotation angle was determined to be $\theta=180^{\circ}$.





**Figure S1.** Average PSNR of the encrypted holographic images as a function of the second distance (a) and second in-plane rotation angle (b) between two cascaded AHLs.

**Section S3: Vulnerability tests of the one-, two-, and three-dimensional acoustic encryption**


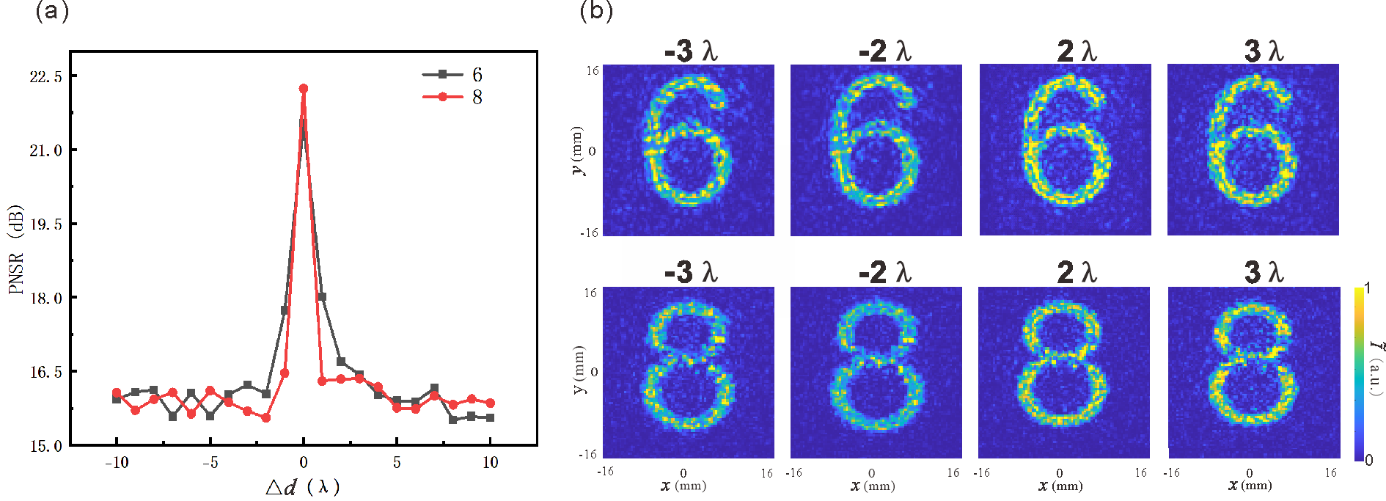


**Figure S2.** Vulnerability test of the one-dimensional acoustic encryption. (a) Average PSNR of the encrypted holographic images as a function of $\Delta d$. (b) Reconstructed acoustic intensity fields under different deviated distances between two cascaded AHLs. Corresponding values above images indicate the deviated distances between two cascaded AHLs.


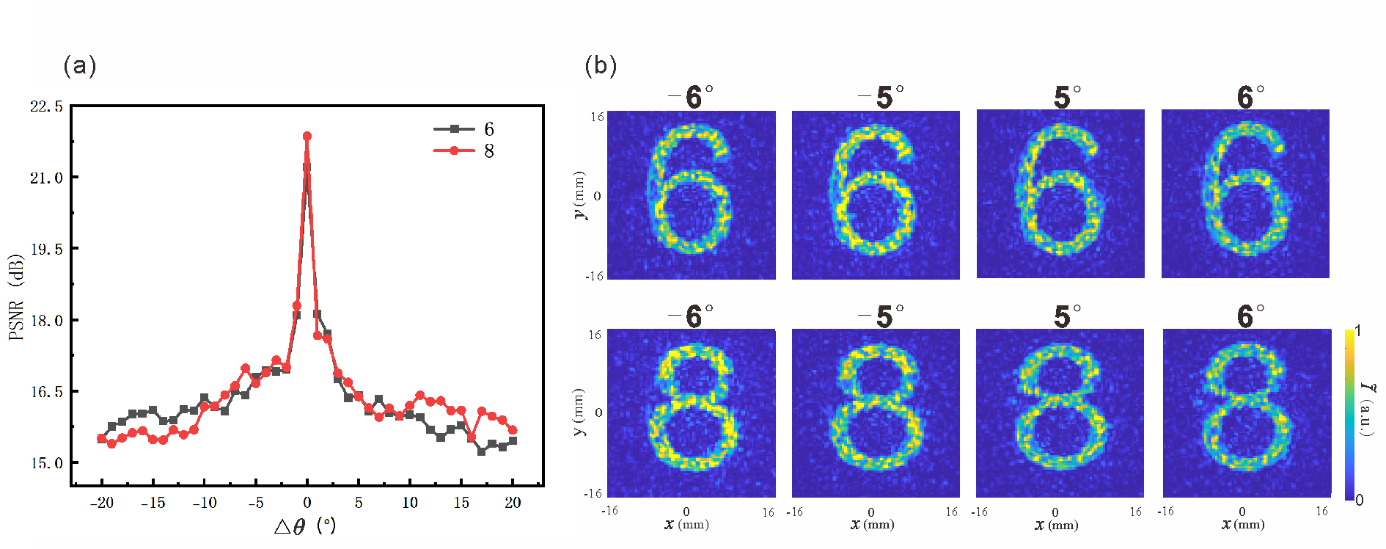


**Figure S3.** Vulnerability test of the two-dimensional acoustic encryption. (a) Average PSNR of the encrypted holographic images as a function of $\Delta\theta$. (b) Reconstructed acoustic intensity fields under different deviated in-plane rotation angles between two cascaded AHLs. Corresponding values above images indicate the deviated in-plane rotation angles between two cascaded AHLs.


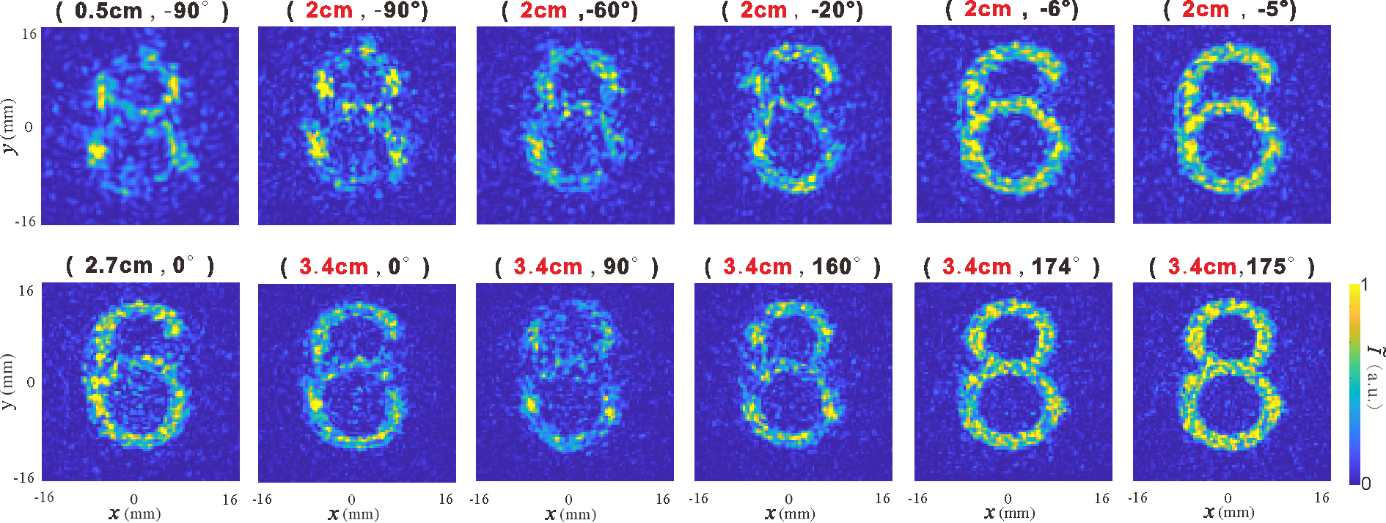


**Figure S4.** Crack difficulty analysis of three-dimensional acoustic encryption. Some intermediate results from the dynamic evolution process of the reconstructed encrypted images as two secret keys are gradually deciphered (Movie S4). Corresponding values above images indicate the distances and in-plane rotation angles between two cascaded AHLs, where the values marked in red indicate that the corresponding secret keys have been correctly deciphered.

**Section S4: Example of three-dimensional acoustic encryption and visual decryption test for letters**

To demonstrate the flexibility and broad applicability of the device, we conducted an additional test of three-dimensional acoustic encryption and visual decryption for the letters “C” and “D”. As presented in Movie S5 and Figure S5, the numerical and experimental results confirm that our VMD-CHAE device can achieve high-quality 3D acoustic encryption and rapid visual decryption of the target letters.


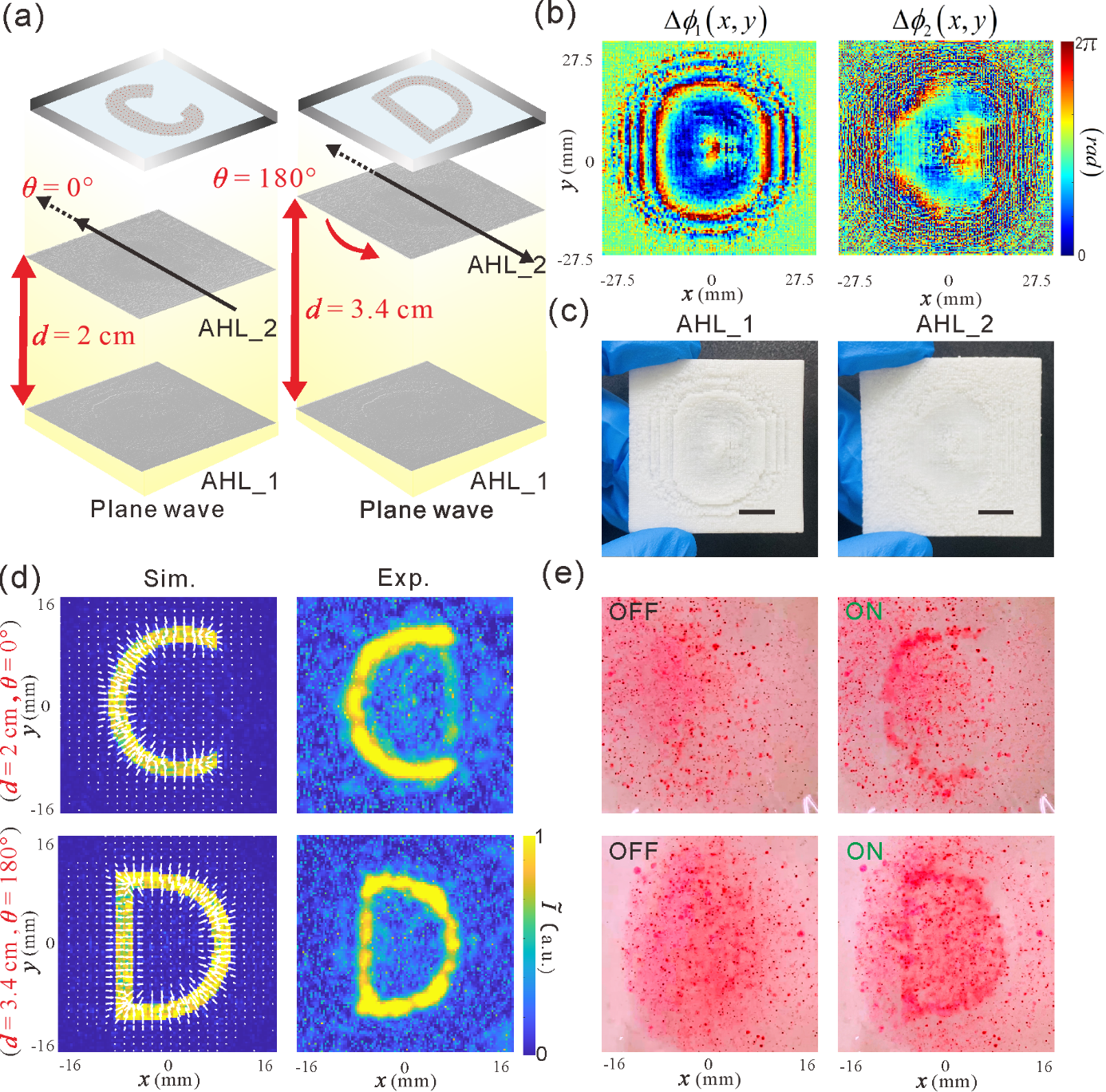


**Figure S5.** Example of three-dimensional acoustic encryption and visual decryption for letters. (a) Design principle and schematics for three-dimensional acoustic encryption and visual decryption via manipulating colored PDMS microparticles. Here, beyond phase profiles, both the distance $d$ and in-plane rotation angle $\theta$ between two cascaded AHLs are used as additional secret keys to encode encrypted holographic images of the letters “C” and “D”, realizing three-dimensional acoustic encryption and visual decryption. (b) Optimized phase profiles of two AHLs. (c) Photograph of the 3D-printed samples of two AHLs. The scale bars in (c) are 110 µm. (d) Simulated and experimental results of encrypted holographic images (acoustic intensity fields), where the length and direction of the white arrows superimposed on the simulated results indicate the relative magnitude and direction of ARFs exerted on the PDMS microparticles, respectively. (e) Experimental results of the visual decryption dynamics of the colored PDMS microparticles when the acoustic power is off（left）and on (right).

**Section S5: Performance comparison for different optimization methods**

To investigate the impact of optimization methods on the device’s performance, we compared the proposed scheme with the modified IASA and the conventional data-driven scheme in three-dimensional acoustic encryption. Since IASA is only suitable for designing a single hologram, we modified it (referred to as modified IASA) for designing cascaded holograms in three-dimensional acoustic encryption. We begin by assigning random phase profiles to the two cascaded AHLs. In each iteration, we propagate forward to the target plane, replace the far-field amplitude with the desired image (first “6”, then “8”) while preserving the phase profiles, and then back-propagate via the angular-spectrum method to update the phase profiles of the two cascaded AHLs. A 180° counterclockwise rotation of the phase profile of AHL_2 in the second sub-loop couples the two constraints and guides convergence. After sufficient iterations, the alternating amplitude-replacement and phase-feedback procedure yields optimized phase profiles that reconstruct both target patterns in the far field. For sufficient convergence, the number of iterations was set to 200.

For the conventional data-driven scheme, it employed the same network structure and physical models as the proposed physics-driven scheme, but was trained in an unsupervised learning manner. The training dataset contains 12,000 image groups, where each group includes 2 target images randomly selected from 12,000 handwritten digits in the Modified National Institute of Standards and Technology (MNIST) database.^[7]^ Among these, 10,000 image groups were used for training, and the remaining 2,000 for validation. The resolution and size of the training images were resized to match those of the encrypted digits “6” and “8”. The loss function and parameters of the Adam optimizer were also set identically to the proposed physics-driven scheme. The mini-batch size was 10, and an early stopping strategy with a patience of 15 was employed to terminate training earlier than the planned 300 epochs.

Table S1 summarizes the quantitative results of three methods in terms of average PSNR, average diffraction efficiency (DE), and computational time for three-dimensional acoustic encryption. Here, DE is defined as the ratio of the total acoustic intensity over the target region to the total incident acoustic intensity. As illustrated in Table S1, the proposed physics-driven scheme achieves the highest performance in the reconstruction accuracy metric (PSNR), which further enables higher-fidelity encryption and superior visual decryption of encrypted information through particle manipulation.

**Table S1.** Quantitative comparison of three methods in terms of average PSNR, average DE, and computational time for three-dimensional acoustic encryption.

| Methods | average PSNR (dB) | average DE | time (s) |
| --- | --- | --- | --- |
| Proposed scheme | 21.31 | 79.81% | 120.13 |
| modified IASA | 15.99 | 82.80% | 10.88 |
| data-driven scheme | 18.79 | 75.61% | 0.05 |

**Section S6: The convergence process of the proposed scheme for the three-dimensional acoustic encryption**





**Figure S6.** The convergence process of the proposed physics-driven scheme for three-dimensional acoustic encryption.


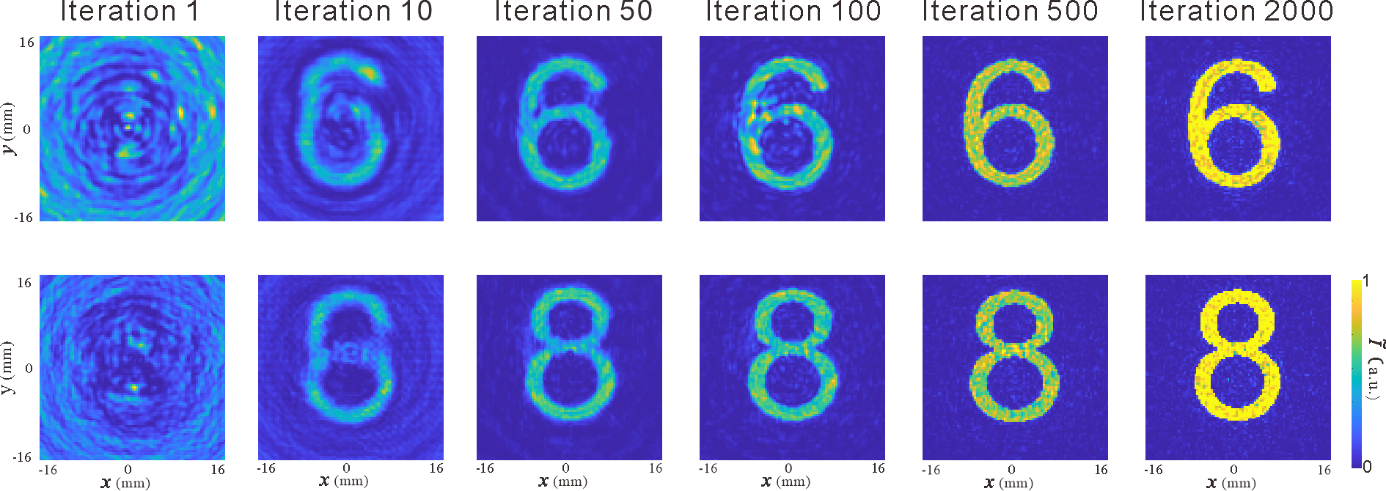


**Figure S7.** Reconstructed intensity fields at selected intermediate iterations of 1, 10, 50, 100, 500 and 2000 of the converge process in Figure S6.

**Section S7: The effects of the number and complexity of encrypted images on the device's performance**

To investigate the effect of effects of the number *N* and complexity of encrypted images, a set of acoustic holograms were calculated, where *N* was varied from 2 to 8. Each case was designed to generate an increasing number of digits (digits “1”and “2” , digits “1” to “4”, digits “1” to “6”, digits “1” to “8”) and letters (letters “A” and “B”, letters “A” to “D”, letters “A” to “F”, letters “A” to “H”), respectively. Different digits (or letters) were encrypted under specific combinations of distances and in-plane rotation angles: digit “1” or letter “A” for ($d=2$cm, $\theta=0^{\circ}$), digit “2” or letter “B” for ($d=2$cm, $\theta=90^{\circ}$), digit “3” or letter “C” for ($d=2$cm, $\theta=180^{\circ}$), digit “4” or letter “D” for ($d=2$cm, $\theta=270^{\circ}$), digit “5” or letter “E” for ($d=3.4$cm, $\theta=0^{\circ}$), digit “6” or letter “F” for ($d=3.4$cm, $\theta=90^{\circ}$), digit “7” or letter “G” for ($d=3.4$cm, $\theta=180^{\circ}$), digit “8” or letter “H” for ($d=3.4$cm, $\theta=270^{\circ}$).


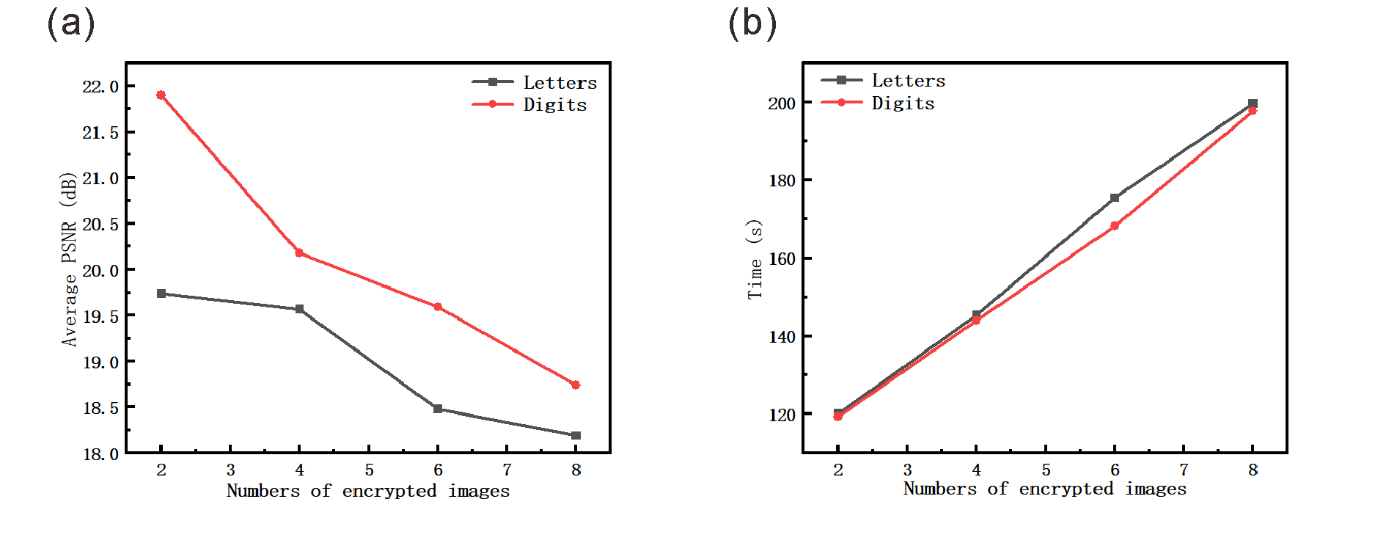


**Figure S8.** (a) Average PSNR of the encrypted holographic images as a function of the number and complexity of encrypted images. (b) Computational time as a function of the number of encrypted images.

**Section S8: The effects of the misalignment and tilt angle between two cascaded AHLs on the device's performance**





**Figure S9.** Average PSNR of the encrypted holographic images as a function of the misalignment (a) and tilted angle (b) between two cascaded AHLs.

**Section S9: Experimental setups for acoustic fields measurement and particle manipulation**


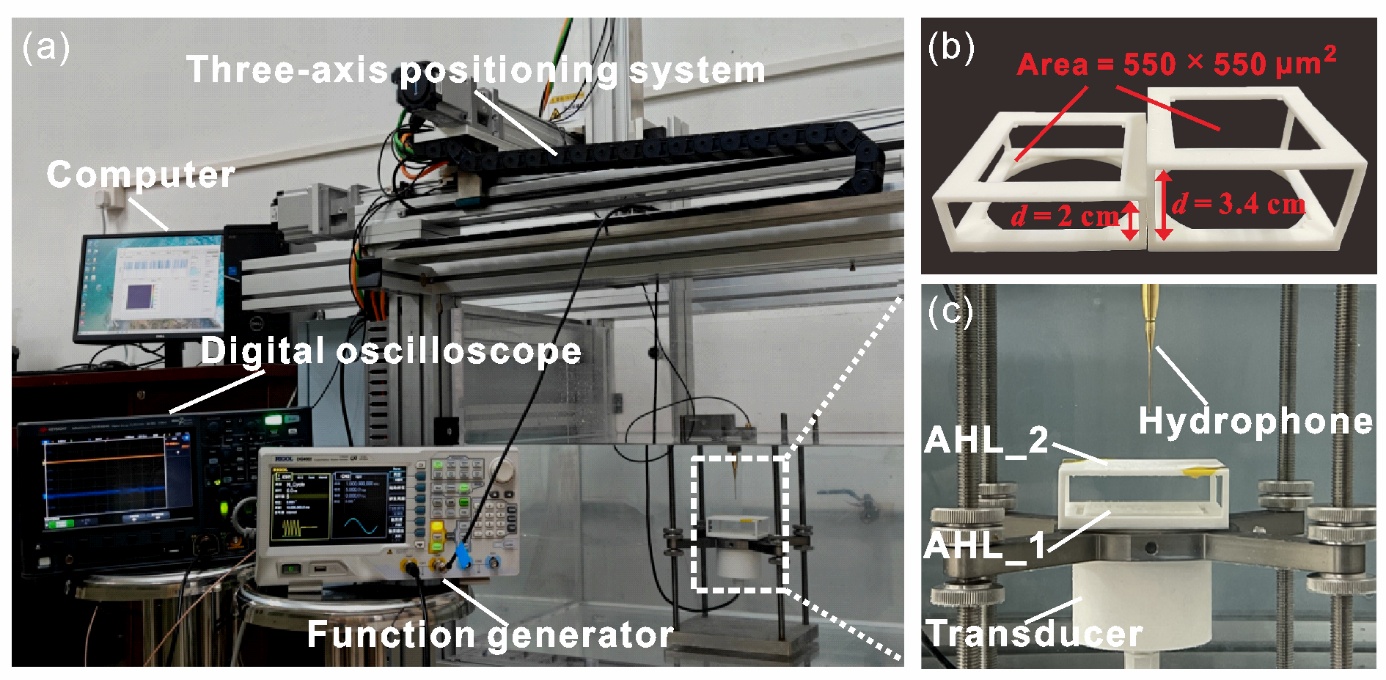


**Figure S10.** (a) Schematic of the experimental setup for pressure fields measurement. (b) Photograph of two 3D-printed two-layer stands. (c) Zooming-in display of the acoustic pressure fields scanning region.


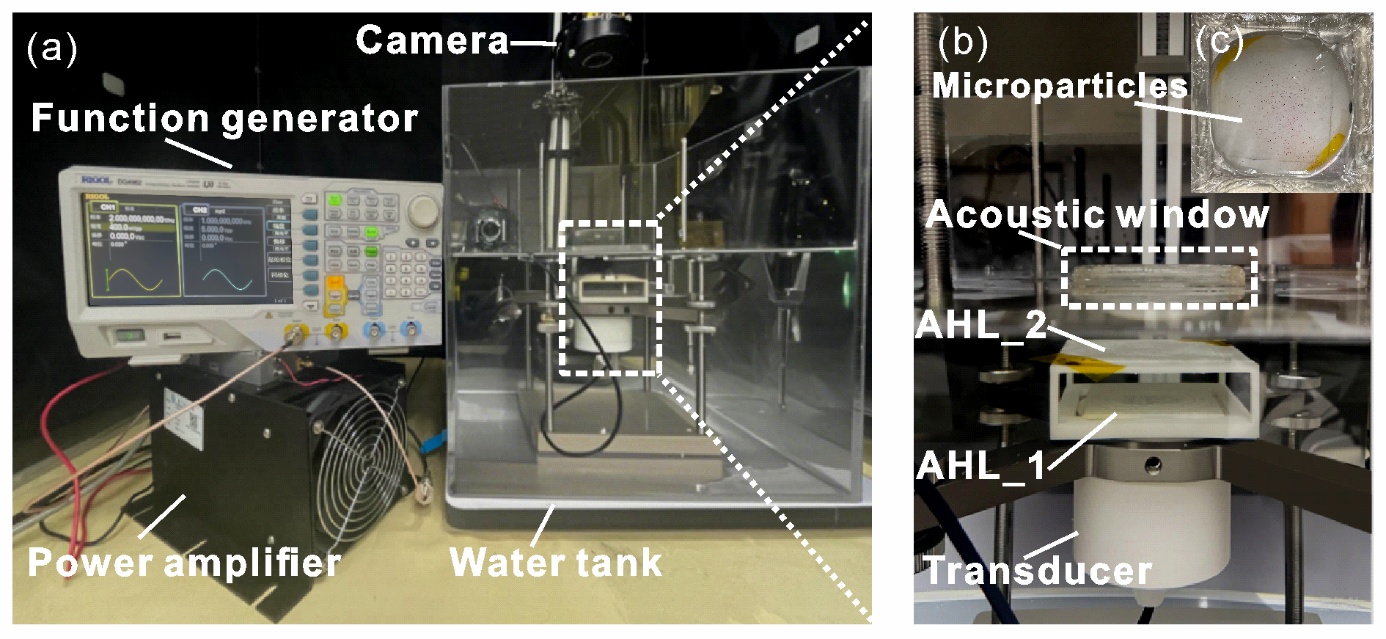


**Figure S11.** (a) Schematic of the experimental setup for particle manipulation. (b) Zooming-in display of the particle manipulation region. (c) Photograph of the acoustic window.

**References**

1. K. Melde, A. G. Mark, T. Qiu et al., “Holograms for acoustics,” *Nature.* 537,no. 7621 (2016): 518-522.

<https://doi.org/10.1038/nature19755>

1. Q. Lin, P. Wang, F. Cai et al., “Deep learning-empowered moving cascaded acoustic holography for high-fidelity and high-capacity acoustic holographic reconstruction,” *Applied Acoustics.* 216,no. (2024): 109768.

[https://doi.org/10.1016/j.apacoust.2023.109768](https://doi.org/https://doi.org/10.1016/j.apacoust.2023.109768)

1. A. G. Athanassiadis, L. Schlieder, K. Melde et al., “Multiplane Diffractive Acoustic Networks,” *IEEE Transactions on Ultrasonics, Ferroelectrics, and Frequency Control.* 70,no. 5 (2023): 441-448.

<https://doi.org/10.1109/TUFFC.2023.3255992>

1. M. D. Brown, B. T. Cox, and B. E. Treeby, “Stackable acoustic holograms,” *Applied Physics Letters.* 116,no. 26 (2020):261901.

<https://doi.org/10.1063/5.0009829>

1. W. Gu, J. Wang, S. Chai et al., “Holographic Reconstruction With All-Acoustic Diffractive Network,” *IEEE Transactions on Computational Imaging.* 10,no. (2024): 129-142.

<https://doi.org/10.1109/TCI.2024.3356867>

1. Q. Lin, R. Zhang, F. Cai et al., “Multi-frequency acoustic hologram generation with a physics-enhanced deep neural network,” *Ultrasonics.* 132,no. (2023): 106970.

[https://doi.org/10.1016/j.ultras.2023.106970](https://doi.org/https://doi.org/10.1016/j.ultras.2023.106970)

1. L. Deng, “The MNIST Database of Handwritten Digit Images for Machine Learning Research,” *IEEE Signal Processing Magazine.* 29,no. 6 (2012): 141-142.

<https://doi.org/10.1109/MSP.2012.2211477>
